# Evaluation of healthcare efficiency in China: a three-stage data envelopment analysis of directional slacks-based measure

**DOI:** 10.3389/fpubh.2024.1393143

**Published:** 2024-05-30

**Authors:** Bingxue Fang, Mincai Li

**Affiliations:** School of Public Health, Sun Yat-sen University, Shenzhen, Guangdong, China

**Keywords:** healthcare efficiency, three-stage data envelopment analysis (DEA), slacks-based measure (SBM), directional distance function (DDF), input-output slacks, China

## Abstract

**Background:**

A consensus on the changing pattern of healthcare efficiency in China is current absent. This study tried to identify temporal fluctuations in healthcare efficiency from 2012 to 2021, and conducted a comparative analysis on the performance of 31 regions in China using region-level balanced panel data.

**Methods:**

Employing three-stage data envelopment analysis (DEA) as the analytical framework, we measured healthcare efficiency and its changes using the directional slacks-based measure and global Malmquist-luenberger (GML) indexes. We also decomposed the sources of healthcare inefficiency and extended our analysis to changes in healthcare efficiency across different primary medical service levels and regional economic development tiers.

**Results:**

The average efficiency score of medical institutions (0.956) was slightly higher than that of hospitals (0.930). We found that the average GML indexes of medical institutions in China stood at 0.990, while the average technical change (TC) index was 0.995 and the average efficiency change (EC) index was 0.998 from 2012 to 2021. The GML indexes, TC indexes, and EC indexes of hospitals were 1.002, 1.009, and 0.994, respectively. The healthcare inefficiency for both inputs and desirable outputs in medical institutions was primarily attributed to the redundant numbers of institutions, outpatient visits slacks and inpatient surgery volume slacks, accounting for 50.040, 49.644, and 28.877%, respectively. The undesirable output inefficiency values of medical institutions concerning in-hospital mortality stood at 0.012, while the figure for hospital regarding the average length of stay (LOS) was 0.002. Additionally, healthcare efficiency in both medical institutions and hospitals exhibited an upward trend from 2012 to 2021, corresponding to an increase in the volume of primary medical services, primary medical staff, and the total gross domestic product (GDP).

**Conclusion:**

Total factor productivity (TFP) of medical services declined in China from 2012 to 2021. The excessive number of medical institutions and the slack of medical service volumes were the main sources of healthcare inefficiency. Regions prioritizing primary medical services and boasting higher GDP levels exhibited superior healthcare efficiency. These findings are expected to inform policymakers' efforts in building a value-based and efficient health service system in China.

## 1 Introduction

Enhancing healthcare efficiency and establishing sustainable healthcare systems poses common challenges for policymakers worldwide. Since 2009, China has embarked on a series of comprehensive reforms, including public hospitals reform, fortification of primary medical institutions, and payment methods reform. However, the extent to which these reforms have improved medical services in China remains uncertain. It is particularly pertinent to investigate which reforms have effectively enhanced the efficiency of medical services during specific time periods. Concurrently, governmental investments in medical domains have also witnessed a significant increase. The data illustrates that a significant increase in China's total health expenditure, surging from CN¥ 1,754.192 billion in 2009 to CN¥ 6,584.139 billion in 2019, with annual growth rates ranging from 11.030 to 17.930%. Concurrently, the proportion of healthcare expenditure to gross domestic product (GDP) rose from 5.150 to 6.670%, while the total health expenditure per capita escalated from CN¥ 1,314.200 to CN¥ 4,669.300. Additionally, there was a significant increase in the number of medical technicians per 1,000 population, rising from 4.152 in 2009 to 7.570 in 2020. Similarly, the number of beds in national medical institutions also experienced substantial growth, climbing from 3.320 in 2009 to 6.460 in 2020 (1). Despite the significant expansions in medical investment, China still faces persistent challenges, including the uneven distribution of high-quality medical resources and weaknesses in the macro-management of healthcare resources allocation (2). Wang and Wei's research findings indicate that despite substantial medical investments, high healthcare efficiency is not guaranteed (3). Given the imperative to maximize the utility of limited resources, there is growing emphasis on the measurement of healthcare efficiency (4).

China is a populous country (5), especially with its population density reaching 150 people per square kilometer. Similar to many middle-income countries, China's healthcare system grapples with increasing strain due to aging population. Characterized by inefficiency and fragmentation, the traditional healthcare systems in China were deemed inadequate to meet the escalating healthcare demand (6). Despite rapid economic development in recent years, the imbalanced economic growth has exacerbated regional and urban-rural disparity in medical service levels (7, 8). To address this issue, several pilot initiatives have been implemented, including medical treatment combination, patient-centered integrated care and hierarchic healthcare (9). However, the impact of these policies on the efficiency and quality of medical services across regions remains uncertain. Healthcare efficiency serves as a vital gauge of a health system's performance (10). Therefore, there is practical significance in examining both overall and regional efficiency of medical services in China. This examination aims to enhance the allocation of medical resources within China and promote the establishment of a high-quality and efficient medical service system.

The development and application of data envelopment analysis (DEA) models for evaluating healthcare efficiency have witnessed remarkable growth. However, previous publications predominantly focused on assessing the efficiency of individual provinces in specific years within the Chinese context. Most studies showed that the healthcare efficiency in China was high and generally had fluctuating upward trends (2, 11, 12). By contrast, Xia et al. found the efficiency of primary medical institutions tended to be low and exhibited regional differences (13). Consequently, there is a lack of consensus regarding the changing trend of medical service efficiency in China. Empirical evidence on Chinese healthcare efficiency evaluation remains limited, posing significant barriers to the development of efficient healthcare systems. The primary objective of this study was to examine temporal fluctuations and regional disparities of medical service efficiency in China's mainland from 2012 to 2021. Additionally, the investigation aimed to identify the origin of healthcare inefficiency by incorporating medical quality indicators. Given the substantial variations in economic development and healthcare system maturity among Chinese provinces, a provincial-level analysis was deemed more effective for understanding healthcare efficiency in China. Additionally, inherent heterogeneity and non-uniformity in studies assessing on the efficiency of decision-making units (DMUs) present challenges in comparing efficiency values across different time periods (14). To address these challenges, this paper employed a three-stage DEA approach that integrates both non-parametric and parametric methods as the analytical framework. Specifically, the analysis utilized the slack-based measure (SBM) with the directional distance function (DDF) and the global Malmquist-luenberger (GML) indexes to evaluate healthcare efficiency across 31 Chinese provinces and cities (hereinafter referred to regions).^1^

This study introduced several innovative methodological advancements. Firstly, it integrated the slack-based measured directional distance function (SBM-DDF) into the traditional three-stage DEA framework, surpassing the conventional DEA model used in the initial and final stages. This integration allowed healthcare inputs and outputs to vary disproportionately (non-radial), eliminating the need to choose between input-based or output-based models during efficiency evaluations (non-guided). Moreover, the second stage of this methodology involved constructing stochastic frontier analysis (SFA) models to neutralize the impacts of external factors across regions, such as environmental variables and stochastic disturbances. Secondly, it incorporated the GML analysis based on the SBM-DDF model to establish a unified production frontier, aligning with the overarching goal of achieving holistic cross-regional comparability within the efficiency evaluation paradigm. Thirdly, the study included an inefficiency analysis to assess the average congestion or deficiency level of specific input and output indicators. Additionally, the study extended its analytical scope beyond medical institutions to encompass hospitals, thereby validating the main analysis outcomes. Undesirable outputs and medical quality indicators were also incorporated into the efficiency evaluation framework.

The remainder of the paper is organized as follows. Section 2 summarizes the literature on healthcare efficiency evaluation. Section 3 systematically introduces the research methodology. Section 4 elaborates on data sources, input-output indicators and environmental variables. Section 5 details the results of main analyses, and describes additional tests conducted in the study, including robustness checks. The results and their implications are discussed in Section 6, where the limitations of the present analysis are also outlined. Finally, Section 7 concludes the paper.

## 2 Literature review

Healthcare efficiency refers to the quantitative correlation between various inputs and outputs within a healthcare delivery system over a specific time period (15). In this paper, healthcare efficiency entails using minimal medical resources to maximize desirable outputs and minimize undesirable ones. Total factor productivity (TFP) is a widely used measure for assessing productivity (16). However, the complexity of multiple inputs and outputs inherent in the medical domain often surpasses conventional cost-benefit frameworks in healthcare analysis (2). Studies typically involve the parameter form given by the stochastic frontier approach (SFA) and the non-parametric method represented by data envelopment analysis (DEA) in terms of healthcare efficiency study approaches (17). The two alternative approaches have different strengths and weaknesses. DEA and related tools like Malmquist indices and distance functions are preferred for analyzing healthcare provider efficiency (18). Michael Farrell constructed a piece-wise linear technology representing the best practice methods of production and then used linear programming to estimate a radial measure of technical efficiency in 1957. Charnes, Cooper, and Rhodes (CCR) (19) and Banker, Charnes, and Cooper (BCC) (20) extended and popularized Farrell's method, naming it DEA (21). Nunamaker first applied the DEA model to medical service domain in 1983 (22), followed by Sherman who used this method to evaluate the multivariate input-output efficiency of American teaching hospitals (23). DEA evaluates the relative efficiency of DMUs based on multiple inputs and outputs, without making assumptions about the functional form of production frontier or inefficiency distribution (24). The choice between input or output orientation remains a question in DEA applications. Charnes et al. addressed this by introducing additive models (ADD) that combine both orientations (25). However, basic DEA models lack the ability to account for slacks in efficiency score, while the ADD model lacks a scalar efficiency measurement (26). To address these limitations, Tone proposed the SBM model (27, 28), which is monotonically decreasing in each slack and provides efficiency measures bounded between zero and one. This model offers a refined approach to assessing efficiency in healthcare settings.

In contrast to Farrell's technical efficiency, Shephard's distance functions were employed to calculate and decompose cost and revenue efficiency (29). As a further extension, DDF was introduced by Luenberger (30). The DDF is a generalization of the input and output distance function (31), whose advantage is the possibility to handle both desirable outputs and undesirable outputs. However, DDF was susceptible to the problem of slack in the technological constraints (21). As mentioned, radial measures of efficiency tend to overestimate technical efficiency in the presence of non-zero slacks in the constraints defining the piece-wise linear technology. To address this issue, researchers have developed alternative efficiency measures that account for slack. Fukuyama and Weber combined the SBM model with DDF to formulate a non-radial and non-oriented SBM-DDF model (21). This innovative approach provides a generalized measure of technical inefficiency by accounting for all slack in the input and output constraints. It enables the evaluations of non-proportional shifts in input-output factors (32).

In panel data analysis, understanding how efficiency values evolve over time is crucial. One contentious issue is whether efficiency changes result from changes in performance of the DMUs themselves, or from shifts of the efficiency frontier. The Malmquist index (MI), combined with DEA, is the preferred tool for panel data analysis (33, 34). MI compared the efficiency of DMUs in one period to the efficiency frontier of another period, thereby creating an intertemporal score (35). However, Ray and Desli highlighted internal inconsistencies in the decomposition of intertemporal effects (36). And environmental effect is an important factor to avoid efficiency measure bias (37). Malmquist-luenberger (ML) productivity index was introduced by Chung et al. (38) to measure environmentally sensitive productivity growth. It integrates the concepts of the Malmquist productivity index and directional distance function. The ML index, however, is not circular and faces a potential linear programming infeasibility problem in measuring cross-period directional distance functions. As an alternative, the GML index constructs the best-practice global technology frontier from the data to circumvent the infeasibility and circularity problem (39).

Compared to the DEA model, SFA, as a one-step estimation method and a specific case of the mixed-effects model, considers stochastic noise in data and enables the statistical testing of hypotheses regarding production structure and inefficiency levels (16). However, SFA has notable weaknesses. It necessitates an explicit imposition of a parametric functional form representing the underlying technology and relies on explicit distributional assumptions for inefficiency terms. The share of studies comparing DEA and the parametric SFA has declined significantly in recent years (40). By contrast, there was a rise in studies employing parametric regression as a second-stage analysis (26). The typical two-stage approach involves conducting a first-stage DEA exercise based on inputs and outputs, followed by a regression analysis in the second stage to explain variations in efficiency scores using observable environmental variables (41). But this evaluation overlooks the impacts of both the operating environment and statistical noise on producer performance. Therefore, employing SFA in the second stage to attribute variation in first-stage producer performance to environmental effects, managerial inefficiency, and statistical noise is a good choice (42). Additionally, the three-stage DEA model adjusts producers' inputs or outputs to account for the environmental effects and statistical noise uncovered in the second stage and then repeats the first-stage analysis by applying DEA to the adjusted data (14).

The application of DEA to investigate healthcare efficiency in China has emerged recently, with traditional DEA methodology remaining prevalent (43). However, scholars are increasingly combining the DEA model with other methods to assess the efficiency of the Chinese healthcare system (44). The scope of healthcare efficiency evaluation diverged between macro and micro levels. The micro perspective entailed an examination of healthcare efficiency within individual medical institutions and hospitals. For instance, Pang evaluated the operation efficiency of 249 hospitals using DEA method and analyzed factors influencing efficiency with a Tobit regression model. This study also introduced medical quality as a factor into the efficiency evaluation model (45). Wang and Pan assessed the operational efficiency of hospitals in Xinjiang Production and Construction Corps by using DEA's CCR model and BCC model (46). Conversely, the macro perspective scrutinized national-scale healthcare efficiency within medical service systems. Yang et al. investigated the technical efficiency and TFP of health resources in Hubei Province from 2012 to 2014 and analyzed factors influencing technical efficiency (47). Zhang et al. measured comprehensive efficiency, pure technical efficiency, and scale efficiency using DEA method and conducted a dynamical comparison of average efficiency in all Chinese regions (48). Additionally, some studies utilized the SFA method to evaluate the efficiency of healthcare delivery systems. For instance, Shen and Zheng employed fixed-effect panel stochastic frontier model to evaluate healthcare efficiency in 2010–2014 and analyzed its influencing factors (49).

The review of previous literature on medical service efficiency in China revealed several research gaps. Firstly, previous studies often assessed the healthcare efficiency across regions under the assumption of the same production technology set. Neglecting technology heterogeneity may lead to biased results (30). Considering China's significant regional gaps, this study examined the healthcare efficiency based on the group heterogeneity using a three-stage DEA model, accounting for environmental effects and statistical noise across regions. Secondly, efficiency changes may be caused by shifts in the non-unified frontier across periods. Most studies overlooked this bias by comparing year-by-year efficiency values. To mitigate this, this study utilized the GML index to compare cross-period efficiency changes, constructing the best-practice global technology frontier from the data. Finally, less attention has been given to examining the undesirable outputs associated with medical services, especially for medical quality. Hence, this study evaluated regional healthcare efficiency in China by incorporating undesirable outputs to provide comprehensive information.

## 3 Methodology

We denoted each production set with (X, Y), in which X represents inputs and Y represents outputs. Under a panel of K DMUs and T time periods, the production technology for medical institutions producing M desirable outputs $y=\left(y_{1},y_{2}\cdots y_{m}\right)\in R_{M}^{+}$ and J undesirable outputs $b=\left(b_{1},b_{2}\cdots b_{j}\right)\in R_{J}^{+}$ by using N inputs $x=\left(x_{1},x_{2}\cdots x_{n}\right)\in R_{N}^{+}$, is represented by the production possibility set (PPS), P(x). (*x*^*k, t*^, *y*^*k, t*^, *b*^*k, t*^) indicates the inputs, desirable outputs and undesirable outputs vector among *DMU*_*k*_ during period t. When relying solely on prevailing production possibility sets, there is a possibility of technological regression. In this regard, we referred to a global PPS introduced by Oh in 2010 (39, 50). This PPS can be specifically expressed as Equation (1):


(1) $$\begin{array}{c} P^{G}(x)=\left\{\begin{array}{c} (y^{t},b^{t}):\sum_{t=1}^{T}\sum_{k=1}^{K}z_{k}^{t}y_{km}^{t}\geq y_{km}^{t},\forall_{m};\sum_{t=1}^{T}\sum_{k=1}^{K}z_{k}^{t}b_{kj}^{t}=b_{kj}^{t},\forall_{j};\\ \sum_{t=1}^{T}\sum_{k=1}^{K}z_{k}^{t}x_{kn}^{t}\geq x_{kn}^{t},\forall_{n};\sum_{t=1}^{T}\sum_{k=1}^{K}z_{k}^{t}=1,z_{k}^{t}\geq 0,\forall_{k}; \end{array}\right\} \end{array}$$

Here, $z_{k}^{t}$ is the weight of each cross-section, while $z_{k}^{t}\geq 0$ means constant return to scale (CRS) and $\overset{K}{\underset{k=1}{\sum }}z_{k}^{t}=1,\;z_{k}^{t}\geq 0$ means variable return to scale (VRS). This global benchmark technology envelopes all contemporaneous benchmark technologies by establishing a single reference PPS from panel data on inputs or outputs of relevant DMUs (39). Additionally, this benchmark technology incorporates undesirable outputs in health production activities.

### 3.1 Three-stage DEA model

This study innovatively replaced traditional DEA model used in the first and third stages of three-stage DEA model with the SBM-DDF model. The adoption of SBM-DDF model cohered with the prerequisites of the three-stage DEA model. In the secondary stage, the SFA model was employed to decompose the slacks of inputs and outputs identified at the primary stage. These slacks encompassed a combination of both radial and non-radial facets, captured by the disparity between original value and target value. This approach encapsulated the redundancy of inputs, undesirable outputs, and the deficiency of desirable outputs. The SBM-DDF model aligns with the pragmatic imperatives inherent in the evaluation of medical services, assuming a pivotal stance of DEA methodologies. The core distinction between SBM-DDF and traditional DEA model lies in that SBM-DDF is based on slack measures and charactered as non-radial and non-guided DDF. The slack-based DEA models also compute target input and output values for inefficient DMUs to identify potential performance improvements (51). The directional distance approach allows for simultaneous output expansion and input contraction (52). Desirable and undesirable outputs could be produced jointly, which is different from traditional DEA model (26). Concurrently, the DDF introduces flexibility by accounting for deviations from the original input-output vector to the production frontier. This enables adjustments in projection direction based on research objectives, rather than being solely constrained to origin-based projection. Considering inputs and outputs of medical systems do not change proportionally in reality, we utilized the SBM-DDF model to estimate healthcare efficiency scores, as illustrated below.

This study evaluated the initial healthcare efficiency of medical institutions using raw panel data and obtained the slack variables corresponding to individual input, desirable output, and undesirable output based on the SBM-DDF model. The global SBM model covering undesirable outputs is defined as follows (21, 53, 54):


(2) $$\begin{array}{l} {\vec{S}}_{\text{​​}v}^{\text{​​}G}\left(x^{t,k^{\prime }},y^{t,k^{\prime }},b^{t,k^{\prime }},g^{x},g^{y},g^{b}\right)=\frac{1}{3}\underset{s^{x},s^{y},s^{b}}{\max}(\frac{1}{N}\sum_{n=1}^{N}\frac{S_{n}^{x}}{g_{n}^{x}}+\frac{1}{M}\sum_{m=1}^{N}\frac{S_{m}^{y}}{g_{m}^{y}}\\ \;+\frac{1}{J}\sum_{j=1}^{I}\frac{S_{j}^{b}}{g_{j}^{b}})\\ \;S.t.\sum_{t=1}^{T}\sum_{k=1}^{K}z_{k}^{t}x_{kn}^{t}+s_{n}^{x}=x_{k^{\prime }n}^{t},\forall_{n};\sum_{t=1}^{T}\sum_{k=1}^{K}z_{k}^{t}y_{km}^{t}-s_{m}^{y}\\ \;=y_{k^{\prime }m}^{t},\forall_{m}; \end{array}$$

$$\begin{array}{l} \sum_{t=1}^{T}\sum_{k=1}^{K}z_{k}^{t}b_{kj}^{t}+s_{j}^{b}=b_{k^{\prime }j}^{t},\forall_{j};\sum_{k=1}^{K}z_{k}^{t}=1,z_{k}^{t}\geq 0,\forall_{k};s_{m}^{y}\geq 0,\\ \;\forall_{m};s_{j}^{b}\geq 0,\forall_{j} \end{array}$$

where (*g*^*x*^, *g*^*y*^, *g*^*b*^) represent positive directional vectors that contract inputs and outputs, while $\left(s_{n}^{x},s_{m}^{y},s_{j}^{b}\right)$ denote slack vectors that inputs and outputs reach at the efficiency frontier. The directional vectors and slack vectors share the same units of measurement as input and output slacks vectors, which enables the addition of normalized slacks. The objective is to maximize the sum of average input inefficiency and average output inefficiency.

In the second stage, SFA model was employed to regress first-stage efficiency measures against a set of environmental variables.^2^ This approach enabled a three-way decomposition of efficiency variation among environmental effects, managerial inefficiency, and statistical noise for each input, desirable output, and undesirable output (depending on the orientation of the first-stage SBM-DDF model). We estimated 10 separate SFA regressions, where dependent variables in the SFA models were the total slacks $\left(s_{n}^{x},s_{m}^{y},s_{j}^{b}\right)$ at stage 1. The independent variables in the SFA regression models are the elements of the 10 observable environmental variables *z*_*i*_ = [*z*_1*i*_, ⋯ , *z*_*Li*_], *i* = 1, …, *I*. The 10 separate SFA regressions take the general form by Equation (3):


(3) $$\begin{array}{l} S_{ni}=f\left(P_{i};\beta_{n}\right)+v_{ni}+u_{ni};i=1,2,\cdots I;n=1,2,\cdots \;,10. \end{array}$$

where *f*(*P*_*i*_; β_*n*_) are deterministic feasible slack frontiers with parameter vectors β_*n*_ to be estimated and composed error structure (*v*_*ni*_+*u*_*ni*_). Consistent with the stochastic cost frontier formulation, we assumed that $v_{ni}\sim N\left(0,\sigma_{vn}^{2}\right)$ reflectd statistical noise and *u*_*ni*_≥0 reflects managerial inefficiency. If we make a distributional assumption on the *u*_*ni*_, such as $u_{ni}\sim N^{+}\left(\mu^{n},\sigma_{un}^{2}\right)$, and if we assume that *v*_*ni*_ and *u*_*ni*_ are distributed independently of each other, and of the *z*_*i*_, each of the 10 regressions (2) may be estimated by maximum likelihood techniques. In each regression, we estimated parameters $\left(\beta_{n},\;\mu_{n},\sigma_{vn}^{2},\sigma_{un}^{2}\right)$, which were allowed to vary across the N slack regressions. This also allows the environmental variables, statistical noise and managerial inefficiency to exert different impacts across inputs and outputs.

The objective of the proposed adjustment is to level medical inputs and outputs for the variable impacts of different operating environments and random statistical noise. One way to level the playing field is to adjust downward the medical inputs and undesirable medical outputs (upward the desirable medical outputs) of these medical institutions, in amounts determined by the extent to which they have been disadvantaged by their relatively unfavorable environments or by their relatively bad luck. The extent to which they have been disadvantaged by each source is revealed by the parameter estimates obtained in the SFA regressions. Another procedure is to adjust upward the inputs and undesirable outputs (downward the desirable outputs) of medical institutions which have been advantaged by their relatively favorable operating environments or by their relatively good luck. We adopted the former approach for desirable outputs adjustments and the latter approach for input adjustments. This choice could avoid the possibility that some extremely disadvantaged medical institutions might have some inputs and outputs adjusted so far as to become negative.^3^ Because the SBM-DDF model in the first stage is non-guided, this study chose to adjust the inputs, desirable outputs and undesirable outputs simultaneously (41). Detailed input-output adjustments were presented in Appendix B.

At stage 3, the observed inputs are replaced with inputs that have been adjusted for the impacts of both environmental variables and statistical noise (41). Utilizing the input-output data adjusted in the second stage and applying the SBM-DDF model, the healthcare efficiency score of each DMU is recalculated.

### 3.2 GML index

The ML index integrate the concepts of the MI and DDF, which has been widely used to measure the performance of DMUs. However, the geometric mean form of ML index is not circular and faces a potential linear programming infeasibility problem when measuring cross-period DDFs. In contrast, the GML productivity index is circular and provides a single measure of productivity change (39). And it constructed the best-practice global technology frontier from the data to circumvent the infeasibility and circularity problem as mentioned above. The GML index, used in this paper,is defined in Equation (4) as follows:


(4) $$\begin{array}{c} GML_{t}^{t+1}\left(x^{t},y^{t},b^{t},x^{t+1},y^{t+1},b^{t+1}\right)=\frac{1+{\vec{S}}_{\text{​​}v}^{\text{​​}G}(x^{t},y^{t},b^{t},g^{x},g^{y},g^{b})}{1+{\vec{S}}_{\text{​​}v}^{\text{​​}G}\left(x^{t+1},y^{t+1},b^{t+1},g^{x},g^{y},g^{b}\right)}\\ =\frac{1+{\vec{S}}_{\text{​​}v}^{\text{​​}t}\left(x^{t},y^{t},b^{t},g^{x},g^{y},g^{b}\right)}{1+{\vec{S}}_{\text{​​}v}^{\text{​​}t+1}\left(x^{t+1},y^{t+1},b^{t+1},g^{x},g^{y},g^{b}\right)}\times \left[\frac{\frac{1+{\vec{S}}_{\text{​​}v}^{\text{​​}G}\left(x^{t},y^{t},b^{t},g^{x},g^{y},g^{b}\right)}{1+{\vec{S}}_{\text{​​}v}^{\text{​​}t}\left(x^{t},y^{t},b^{t},g^{x},g^{y},g^{b}\right)}}{\frac{1+{\vec{S}}_{\text{​​}v}^{\text{​​}G}\left(x^{t+1},y^{t+1},b^{t+1},g^{x},g^{y},g^{b}\right)}{1+{\vec{S}}_{\text{​​}v}^{\text{​​}t+1}\left(x^{t+1},y^{t+1},b^{t+1},g^{x},g^{y},g^{b}\right)}}\right]\\ =\frac{TE^{t+1}}{TE^{t}}\times \frac{BPG_{t+1}^{t,t+1}}{BPG_{t}^{t,t+1}}=EC^{t,t+1}\times TC^{t,t+1}. \end{array}$$

Where the DDF is defined on the global technology set *P*^*G*^(*x*). If a production activity enables more (less) desirable outputs and less (more) undesirable outputs, then $GML_{t}^{t+1}\;>\left(<\right)1$, indicating productivity gain (loss). *TE*^*t*^ is a measure of technical efficiency at time period t. $BPG_{t}^{t,t+1}$ is a best practice gap between contemporaneous technology frontier and global technology frontier, along the ray from the observation at time period t in direction (*g*^*y*^, *g*^*b*^). The efficiency change term, *EC*^*t, t*+1^, is a change in technical efficiency during two period, capturing how close a DMU moves toward a contemporaneous benchmark technology at time period t + 1 compared to time period t. The technical change term, *TC*^*t, t*+1^ measures a shift in contemporaneous benchmark technology frontier. Change in productivity is determined by the simultaneous effect of these two changes. This study utilized technical change (TC) index and efficiency change (EC) index to measure the medical technical improvement and medical technical efficiency changes during the two periods.

### 3.3 Inefficiency value decomposition

The inefficiency value calculated by Equation (2) was further decomposed to identify the specific source of inefficiency in Equation (5):


(5) $$\begin{array}{c} IE=\vec{S_{v}^{t}}=IE_{v}^{x}+IE_{v}^{y}+IE_{v}^{b}. \end{array}$$

$IE_{v}^{x}$, $IE_{v}^{y}$, and $IE_{v}^{b}$ represents the inefficiency values of medical inputs, desirable medical outputs and undesirable medical outputs, respectively (32, 55, 56). They can be calculated by the following Equations (6–8). The detailed decomposition process is attached at the Appendix C.


(6) $$\begin{array}{l} IE_{v}^{x}=\frac{1}{3N}\overset{N}{\underset{n=1}{\sum }}\frac{S_{n}^{x}}{g_{n}^{x}}. \end{array}$$

(7) $$\begin{array}{l} IE_{v}^{y}=\frac{1}{3M}\overset{N}{\underset{m=1}{\sum }}\frac{S_{m}^{y}}{g_{m}^{y}}. \end{array}$$

(8) $$\begin{array}{l} IE_{v}^{b}=\frac{1}{3J}\overset{J}{\underset{j=1}{\sum }}\frac{S_{j}^{b}}{g_{j}^{b}}. \end{array}$$

### 3.4 Hierarchical analysis

The study employed a quartile stratification method to categorize 31 regions in China into three distinct groups: low-level (Q1), middle-level (Q2 and Q3), and high-level (Q4) groups.^4^ To conduct a comprehensive analysis of changes in healthcare efficiency across different levels of primary healthcare service and regional economic development, the study included three key criteria: the proportion of primary service volume (*Service*_*ph*_), the proportion of primary medical staff (*Staff*_*ph*_), and the GDP. We used the level form of *Staff*_*ph*_ and *Service*_*ph*_, which is defined as Equations (9, 10):


(9) $$\begin{array}{l} {\text{Service}}_{\text{ph}}=\frac{{\text{service}}_{\text{primary medical institutions}}}{{\text{service}}_{\text{hospitals}}}. \end{array}$$

Where *service*_*ph*_ represents the proportion of primary service volume, *service*_*primary medical institutions*_ refers to the total number of outpatient visits and hospitalizations provided by primary medical institutions, and *service*_*hospitals*_ represents the total number of outpatient visits and hospitalizations provided by hospitals.


(10) $$\begin{array}{l} {\text{Staff}}_{\text{ph}}=\frac{{\text{staff}}_{\text{primary medical institutions}}}{\text{staf}{\text{f}}_{\text{hospitals}}}. \end{array}$$

Where *Staff*_*ph*_ represent the proportion of primary medical staff. *Staff*_*primary medical institutions*_ denote the total number of doctors and registered nurses in primary medical institutions, and *staff*_*hospitals*_ represent the total number of doctors and registered nurses in hospitals. It is crucial to emphasize that doctors specifically include licensed (assistant) physicians here.

This study employed MATLAB R2018a to evaluate efficiency using the SBM-DDF model developed by Fukuyama and Weber (21). Additionally, FRONTIER software Version 4 was utilized to enable a three-way decomposition of efficiency variation, accounting for environmental effects through the SFA model (57).

## 4 Data and variable selection

### 4.1 Data source

This study utilized region-level healthcare data, encompassing 31 regions in China's mainland. These regions were categorized into three districts based on geographical differences: (1) the eastern district covering 11 regions (Beijing, Tianjin, Hebei, Liaoning, Shanghai, Jiangsu, Zhejiang, Fujian, Shandong, Guangdong, and Hainan); (2) the western district containing 12 regions (Inner Mongolia, Guangxi, Chongqing, Sichuan, Guizhou, Yunnan, Tibet, Shaanxi, Gansu, Qinghai, Ningxia, and Xinjiang); (3) the central district involving eight regions (Shanxi, Jilin, Heilongjiang, Anhui, Jiangxi, Henan, Hubei, and Hunan). Data was directly derived from the China Statistical Yearbook, China Health Statistical Yearbook, and China Population and Employment Statistical Yearbook (1, 58, 59). The research period spanned from 2012 to 2021, excluding 2020. The choice of study years was driven by data availability constraints and the disruption due to the COVID-19 pandemic. Inputs, desirable outputs, undesirable outputs, and environment variables were extracted from medical institutions across all 31 regions, and the data were subsequently stratified by hospitals.

### 4.2 Input-output variables

Efficiency measurement hinges on the selection of appropriate input-output indicators. Choosing suitable inputs and outputs is crucial for accurately characterizing the analyzed process (11). Inputs should incorporate all necessary resources, while outputs should align with the managerial objectives of DMUs (26). Following the service-oriented approach, the selected input variables pertain to the level of activity within medical institutions. The inputs to medical service system in China primarily include labor and capital (2, 3). Healthcare providers, such as physicians and nurses, play a direct role in delivering medical services to patients and collectively influence healthcare outputs (60). Additionally, health cost serve as a pivotal input indicator (61–63). Therefore, this study examined the allocation of medical resources within each DMU based on three categories: medical facilities, personnel, and costs.^5^ Specifically, medical facilities encompass the number of medical institutions (*X*_1_) and beds (*X*_2_). Medical personnel include the number of doctors (*X*_3_) and registered nurses (*X*_4_). Medical costs denote the total costs of medical institutions (*X*_5_).

In existing healthcare literature, outputs are typically considered in terms of staff-oriented activities such as the number of separations. In China, medical institutions and hospitals predominantly focus on outpatient and inpatient care (64). Consequently, within the Chinese context, most studies involving output variables primarily quantify outpatient visits and inpatient discharges. This study evaluated the magnitude of healthcare outputs through both medical service volume and corresponding total income. These variables were categorized by outpatient services and inpatient services, including the number of outpatient visits (*Y*_1_), hospitalizations (*Y*_2_), surgeries (*Y*_3_), and the total income of medical institutions (*Y*_4_).

It is noteworthy that existing studies tend to underestimate the importance of medical quality within the framework of efficiency evaluation. Inpatient quality variables serve as indicators of the healthcare quality provided and typically include mortality rates and readmission rates (4). Due to data availability constraints, this study only considered in-hospital mortality rate (*Y*_5_) as an undesirable output among medical institutions. Similarly, the average length of stay (LOS) and bed occupancy rate were considered as undesirable output variables among hospitals. Based on selection principles, research objectives, healthcare conceptualization, and data availability, this study identified potential input variables, desirable output variables, and undesirable output variables. The total number of inputs and outputs combined does not exceed the amount of DMUs (26). Table 1 and Supplementary Table 1 list input-output variables and their definitions among medical institutions and hospitals. The alignment of these variables is crucial for deriving meaningful efficiency values. In assessing the selected inputs and outputs, we conducted a homogeneity test and a Pearson correlation analysis, which measures the direction and strength of the association among the efficiency measures. The results of the correlation analysis revealed strong correlation between the inputs and outputs (Supplementary Table 8). It is important to note that the correlation of two variable is an aggregate measure over the entire sample size. As such, a high correlation between inputs or outputs is no reason for omitting one of them (51, 52, 65, 66). Ultimately, we included five inputs, four desirable outputs, and one undesirable output among medical institutions and hospitals.

**Table 1:** Inputs and outputs indicators of healthcare efficiency among medical institutions in China.

**Categories**	**Dimension**	**Variables**
Inputs	Medical facilities	Number of medical institutions (*X*_1_)
		Number of beds (*X*_2_)
	Medical personnel	Number of doctors (*X*_3_)
		Number of registered nurses (*X*_4_)
	Medical expenditure	Total expenditure of medical institutions (*X*_5_)
Desirable outputs	Outpatient services	Number of outpatient visits (*Y*_1_)
	Inpatient services	Number of hospitalizations (*Y*_2_)
		Inpatient surgery volumes (*Y*_3_)
	Medical income	Total income of medical institutions (*Y*_4_)
Undesirable outputs	Inpatient quality	In-hospital mortality rates (*Y*_5_)

### 4.3 Environment variables

The selection of environmental variables was primarily based on their potential to impact input slacks rather than their inherent units (67). Health outcomes of DMUs depend on various environmental conditions, including socio-cultural, economic and political factors, many of which are not well-understood or are beyond the control of the healthcare sector. Factors such as regional economic development, governmental health investment, population demographics, and public healthcare utilization were generally considered to influence healthcare efficiency scores in China (48, 49).This study incorporated 10 environmental variables into the analytical framework to assess the efficiency of China's medical services (Table 3). Variables such as regional gross domestic product (GDP) per capita (*P*_1_), the proportion of urban population (*P*_2_), and population density (*P*_3_) were included to reflect regional economic development, while financial subsidy income of medical institutions (*P*_4_) served as an indicator of governmental medical investment in China. Additionally, average years of education (*P*_5_), the proportion of the older adults aged 65 and above (*P*_6_), and mortality rate (*P*_7_) were adopted to reflect population demographics and health status. The rate of basic medical insurance participation (*P*_8_), as well as the per capita healthcare cost for urban residents (*P*_9_) and rural residents (*P*_10_), was applied to reflect public awareness and capability regarding healthcare utilization. This incorporation aimed to provide a more comprehensive and robust assessment of healthcare efficiency by mitigating the impact of uncontrollable factors. Environmental variables were represented using dummy variables, with little attention given to the inherent unit of measurement for these variables (41).

Input and output indicators were converted into intensity vectors by dividing them by the per-unit-population number of each region in the respective year. This process improved the comparability of healthcare input-output resources across various regions nationwide. In addition, the current study adopted non-dummy environmental variables, necessitating their standardization of units to enhance outcome precision. We rescaled the original value of environmental variables within the range of 0 to 20 by appropriate 1,000-fold divisions. These adjustments allowed for the proportional expansion or reduction of impacts exerted by environmental variables (68), thereby not affecting the effect of environmental variables on healthcare efficiency. And cost-related data was adjusted to 2011 to account for inflation, using the annual healthcare consumer price index (CPI) of China.

## 5 Results

### 5.1 Descriptive analysis of input-output and environmental variables

The summary descriptive statistics of input-output variables among medical institutions and environmental variables are displayed in Tables 2, 3. Detailed descriptive results of hospitals could be found in Supplementary Table 2. Additionally, the average growth rate of each input-output variable among medical institutions and hospitals is depicted in Supplementary Table 3. Notably, there was a consistent upward trend in all variables among medical institutions from 2012 to 2021. By contrast, the hospital bed occupancy rate and LOS decreased 2.143 and 1.203%, while other variables exhibited annual increases among hospitals. It was worth noting that in 2020, the total number of outpatient visits, inpatient discharges, and surgeries significantly decreased due to the COVID-19 pandemic among both medical institutions and hospitals.

**Table 2:** Descriptive statistics of input-output variables among medical institutions.

**Categories**	**Variables**	**Sample size**	**Mean**	**Standard deviation**	**Minimum**	**Maximum**
Inputs	*X*_1_	310	7.611	3.301	2.013	21.210
	*X*_2_	310	24.780	5.806	12.830	51.400
	*X*_3_	310	26.950	7.590	5.498	56.690
	*X*_4_	310	55.350	11.140	26.510	83.370
	*X*_5_	310	2,065.000	1,535.000	621.400	13,896.000
Desirable outputs	*Y*_1_	310	550.100	179.000	268.100	1,136.000
	*Y*_2_	310	1,557.000	359.300	457.300	2,420.000
	*Y*_3_	310	381.800	165.400	74.010	1,614.000
	*Y*_4_	310	2,125.000	1,408.000	697.300	9,780.000
Undesirable outputs	*Y*_5_	310	0.423	0.328	0.050	1.700

**Table 3:** Descriptive statistics of environmental variables in 2012–2021.

**Variables**	**Symbols**	**Unit**	**Sample size**	**Mean**	**Standard deviation**	**Minimum**	**Maximum**
GDP per capita	*P*_1_	10,000 yuan	310	5.945	2.869	1.910	18.396
The proportion of urban population	*P*_2_	‰	310	5.902	1.287	2.281	8.960
Population density	*P*_3_	1,000 people/sq km	310	2.854	1.130	1.032	5.541
Financial income	*P*_4_	Ten billion yuan	310	1.793	1.432	0.101	10.806
Average years of education	*P*_5_	Year	310	8.938	0.924	4.556	12.701
The older adults	*P*_6_	%	310	10.905	2.766	4.984	18.805
Mortality rate	*P*_7_	‰	310	6.259	0.928	4.260	8.890
Insurance	*P*_8_	%	310	0.956	0.094	0.710	1.159
Healthcare cost_ urban residents	*P*_9_	%	310	7.521	1.872	3.000	12.800
Healthcare cost_ rural residents	*P*_10_	%	310	9.488	2.359	1.900	15.400

(1) Financial income refers to financial subsidy income of medical institutions.

(2) The older adults refers to the proportion of the older adults aged 65 and above.

(3) Insurance refers to the rate of basic medical insurance participation.

(4) Healthcare cost_urban and healthcare cost_rural refers to the per capita healthcare cost for urban residents and rural residents, separately.

### 5.2 Healthcare efficiency across regions in China

#### 5.2.1 Measurement results of healthcare efficiency

Efficiency performances were presented by scores ranging from 0 to 1, where a score of one indicated full efficiency and a score below one indicated inefficiency. The average efficiency value for medical institutions over the 9-year period was slightly exceeded that of hospitals (0.956 vs. 0.930). Detailed data can be found in Table 4. Notably, the efficiency scores of medical institutions in the eastern, western, and central districts were 0.964, 0.959, and 0.941, respectively, while the corresponding scores for hospitals in these three districts were 0.936, 0.933, and 0.917. The eastern districts exhibited the highest average efficiency scores compared with the other two districts.

**Table 4:** Efficiency scores among medical institutions and hospitals in China and three districts between 2012 and 2021.

**Year**	**Medical institutions**				**Hospitals**			
	**China**	**Eastern districts**	**Western districts**	**Central districts**	**China**	**Eastern districts**	**Western districts**	**Central districts**
2012	0.968	0.976	0.976	0.947	0.925	0.919	0.943	0.905
2013	0.970	0.977	0.975	0.953	0.932	0.936	0.934	0.923
2014	0.969	0.976	0.971	0.954	0.963	0.969	0.966	0.952
2015	0.951	0.965	0.948	0.935	0.934	0.931	0.946	0.921
2016	0.951	0.963	0.946	0.939	0.924	0.940	0.923	0.904
2017	0.944	0.954	0.944	0.931	0.936	0.944	0.938	0.923
2018	0.939	0.948	0.936	0.933	0.909	0.912	0.913	0.900
2019	0.965	0.965	0.973	0.952	0.921	0.930	0.918	0.914
2021	0.948	0.950	0.962	0.924	0.925	0.944	0.917	0.912
Mean	0.956	0.964	0.959	0.941	0.930	0.936	0.933	0.917

Supplementary Table 4 presents the healthcare efficiency of medical institutions across various regions in China from 2012 to 2021 based on the SBM-DDF model. The study identified top 10 regions with the highest average value as “high healthcare efficiency regions.” These regions included Fujian, Jiangsu, Shanghai, Yunnan, Jiangxi, Hunan, Zhejiang, Anhui, Guangdong, and Guizhou. Among these regions, Shanghai, Yunnan, Hunan, Zhejiang, Guangdong, and Guizhou were common to the “high healthcare efficiency regions” of both medical institutions and hospitals. Notably, three of these regions were situated in eastern China, two in western China, and one in central China. The conclusion verifies the robustness of changing trends in Table 4. The healthcare efficiency value and its standard deviation among the eastern district, western district, and central district in 2012–2021 are displayed in Supplementary Table 7. The standard deviation of medical institution efficiency value among three districts increased by an average of 4.838, 3.672, and 4.516%, respectively. The results indicated that the regional gap among the three districts was widening year by year.

#### 5.2.2 Evolution trend of healthcare efficiency

**Figure 1:**
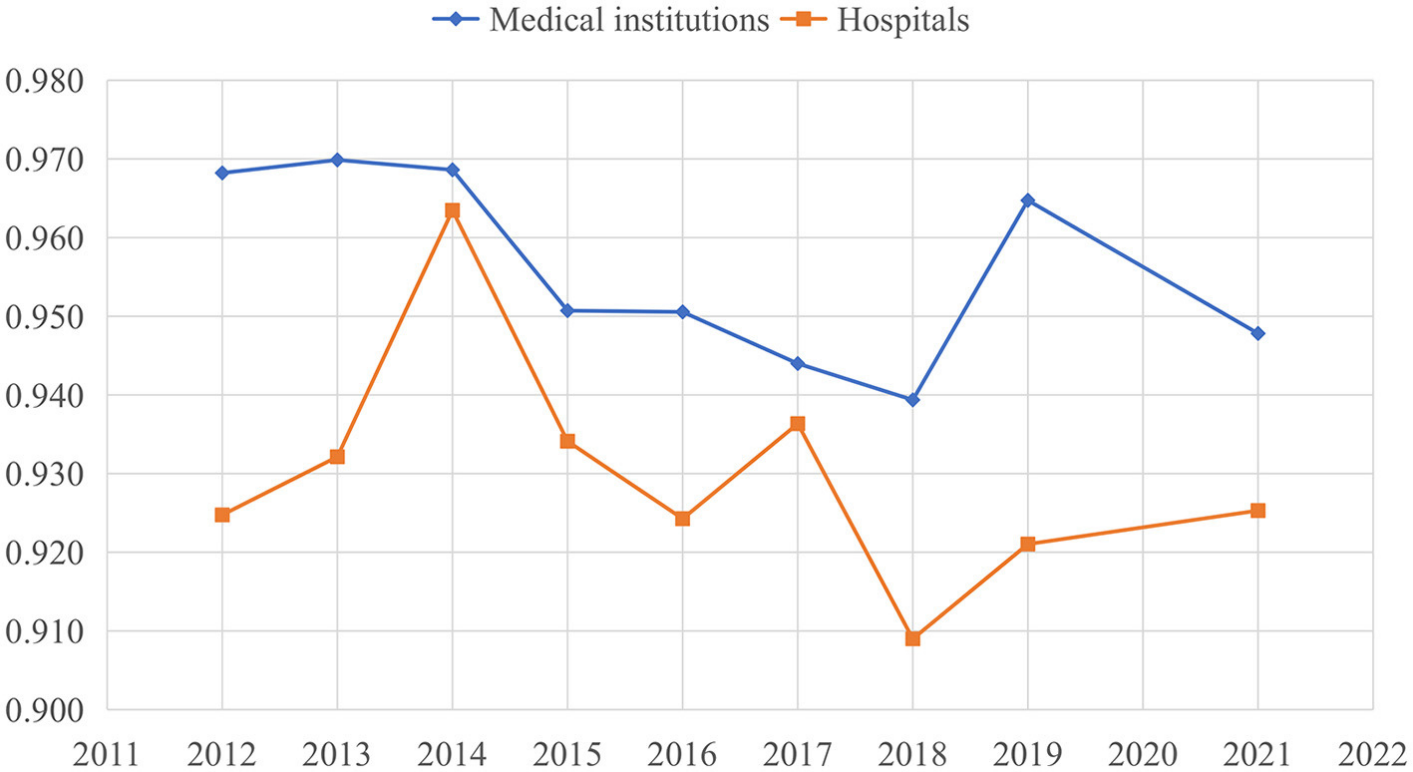
Trend change of healthcare efficiency among medical institutions and hospitals in China.

**Figure 2:**
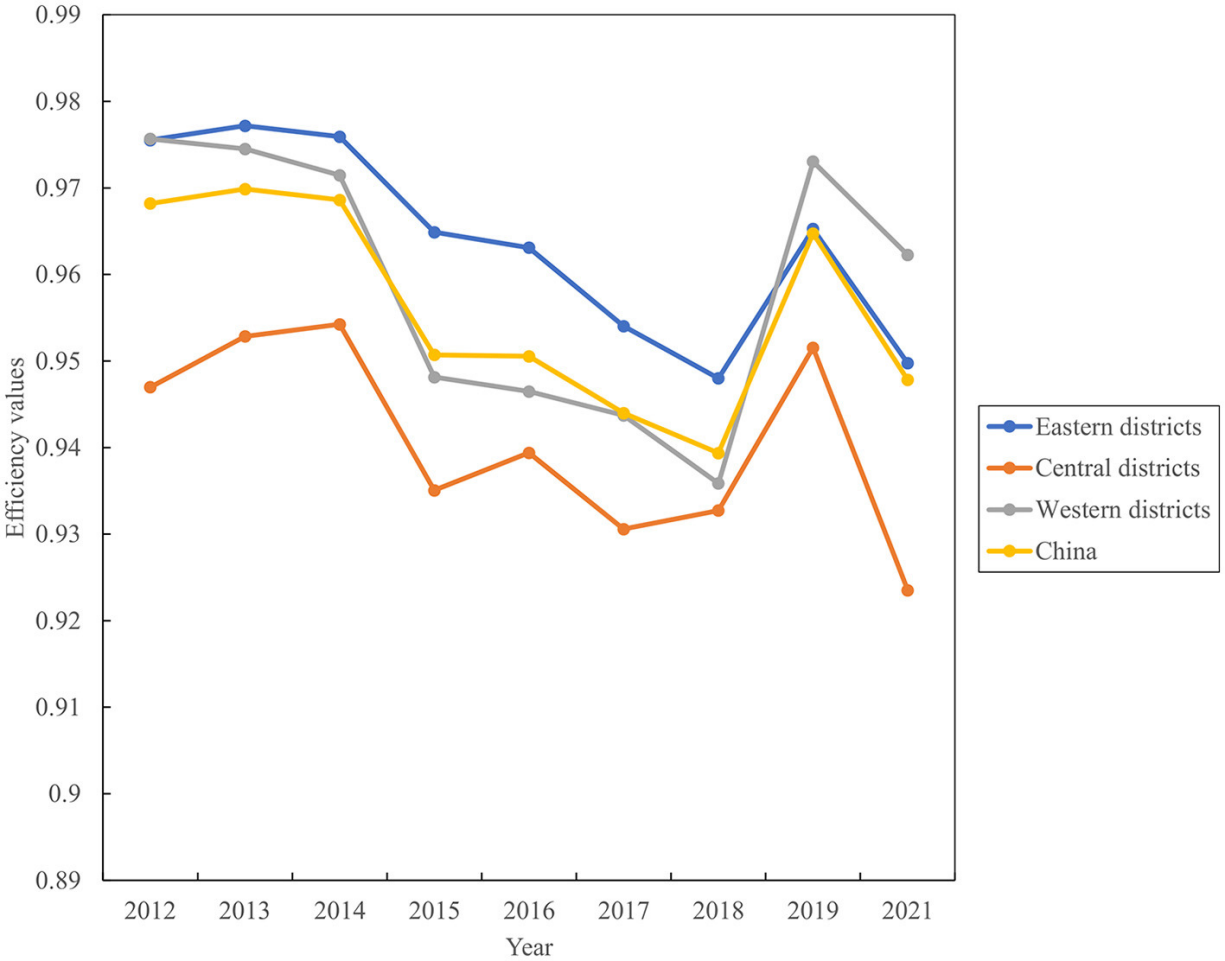
Evolution trend of healthcare efficiency among medical institutions from 2012 to 2021.

Figure 1 illustrates the trend change of China's healthcare efficiency among medical institutions and hospitals from 2012 to 2021. Healthcare efficiency in China fluctuated over this period. Efficiency scores among medical institutions exhibited a downward trend from 2012 to 2018, followed by a rapid increase in 2019 and a subsequent decline in 2021. Hospital efficiency experienced significant increases in 2014 and 2017 (Supplementary Figure 1). Figure 2 compares the changing trends of medical institutions among the eastern districts, western districts, and central districts. The trend of efficiency values in the central districts was consistently lagged behind those in the other two districts in China.

#### 5.2.3 Inefficiency analysis of inputs and outputs

**Figure 3:**
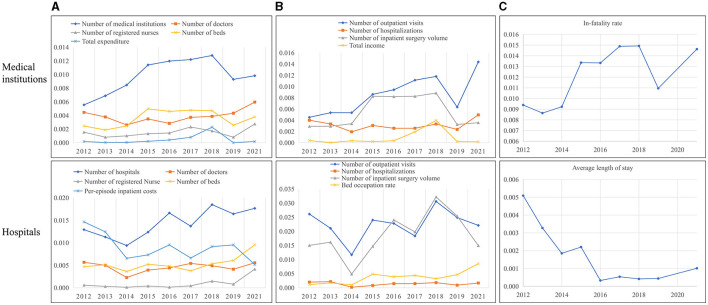
Inefficiency analysis of China's medical service among input-output indicators in 2012–2021. The input and desirable output indicators here are in units of per 10,000 population. **(A)** Input inefficiencies. **(B)** Desirable output inefficiencies. **(C)** Undesirable output inefficiencies.

This study calculated the input-output inefficiency among medical institutions and hospitals in 2012–2021 and reported the results in Table 5 and Supplementary Table 5. The per-unit-population numbers of medical institutions and hospitals were the main source of input inefficiency, which stood at 0.010 and 0.014, respectively. The outpatient visits (49.644 vs. 48.590%) and inpatient surgery volume (28.877 vs. 40.347%) were the common reason for desirable output inefficiency among both medical institutions and hospitals. The undesirable output inefficiency among medical institutions was 0.012, which was chiefly attributed to the annual slack in in-hospital mortality rates by 0.088 percentage points in 2012–2021 (Supplementary Table 6). In contrast, there was a reduction trend in the average LOS concerning the undesirable output inefficiency among hospitals (Figure 3). And the slack of LOS in hospitals decreased from 0.57 days in 2012 to 0.11 days in 2021. The slack values of each input and output in medical institutions and hospitals are listed in Supplementary Table 6.

**Table 5:** The input-output inefficiency scores among medical institutions between 2012 and 2021.

**Year**	**Input variables**					**Desirable output variables**				**Undesirable output variable**
	**X_1_**	**X_2_**	**X_3_**	**X_4_**	**X_5_**	**Y_1_**	**Y_2_**	**Y_3_**	**Y_4_**	**Y_5_**
2012	0.006	0.004	0.002	0.002	0.000	0.005	0.004	0.003	0.000	0.009
2013	0.007	0.004	0.001	0.002	0.000	0.005	0.003	0.003	0.000	0.009
2014	0.008	0.003	0.001	0.002	0.000	0.005	0.002	0.003	0.000	0.009
2015	0.011	0.003	0.001	0.005	0.000	0.009	0.003	0.008	0.000	0.013
2016	0.012	0.003	0.001	0.005	0.000	0.009	0.003	0.008	0.000	0.013
2017	0.012	0.004	0.002	0.005	0.001	0.011	0.003	0.008	0.002	0.015
2018	0.013	0.004	0.002	0.005	0.002	0.012	0.003	0.009	0.004	0.015
2019	0.009	0.004	0.001	0.003	0.000	0.006	0.002	0.003	0.000	0.011
2020	0.010	0.006	0.003	0.004	0.000	0.014	0.005	0.004	0.000	0.015
2021	0.006	0.004	0.002	0.002	0.000	0.005	0.004	0.003	0.000	0.009
Mean	0.010	0.004	0.002	0.004	0.000	0.009	0.003	0.005	0.001	0.012

### 5.3 Analysis of GML indexes and its decomposition

#### 5.3.1 GML indexes and its decomposition at the regional level

Table 6 provides a summary of GML indexes, TC indexes and EC indexes among medical institutions and hospitals across 31 regions in China from 2012 to 2021. The average GML index of medical institutions was 0.990, followed by the average TC index of 0.995 and the average EC index of 0.998 in China. These results indicate that healthcare TFP among medical institutions decreased by an average of 1% between 2012 and 2021 in China. Additionally, medical technology and technical efficiency witnessed an average degradation of 0.5 and 0.2%, respectively. In contrast, the GML index, TC index and EC index among hospital was 1.002, 1.009, and 0.994. The results suggest that healthcare TFP among hospitals improved by 2% from 2012 to 2021. This increase was primarily contributed by the improvement of medical technology (9%). The growth of TPF among medical institutions and hospitals across regions was primarily driven by TC indexes, as shown in Table 6. Specifically, five regions, including Qinghai, Beijing, Hubei, Hunan, and Chongqing, exhibited an upward trend in TPF among medical institutions. The growth of TPF in these regions was driven by TC, with the exception of Qinghai. Additionally, there were 13 regions where hospitals boasted an average GML index exceeding 1. These regions included Beijing, Shaanxi, Henan, Shandong, Tianjin, Shanghai, Hunan, Liaoning, Chongqing, Shanxi, Jiangsu, Ningxia, and Anhui. Similarly, TC was the driving force behind the growth of TFP in these provinces except for Anhui. Detailed sorting data of the GML indexes, TC indexes and EC indexes for each region could be found in Supplementary Table 9.

**Table 6:** TFP changes and the decomposition effect of medical service in China.

**Time period**	**Medical institutions**			**Hospitals**		
	**GML**	**TC**	**EC**	**GML**	**TC**	**EC**
2013/2012	1.002	1.001	1.002	1.009	1.003	1.006
2014/2013	0.999	0.991	1.009	1.035	1.028	1.007
2015/2014	0.982	0.998	0.986	0.969	0.986	0.984
2016/2015	1.000	1.000	1.000	0.990	0.987	1.003
2017/2016	0.993	0.994	0.999	1.013	1.016	0.997
2018/2017	0.995	0.995	1.001	0.971	0.974	0.996
2019/2018	1.027	1.030	0.998	1.014	1.032	0.982
2021/2020	0.983	0.981	1.001	1.005	0.993	1.012
Mean	0.990	0.995	0.998	1.002	1.009	0.994

GML = TC^*^EC.

GML, global Malmquist_luenberger indexes; EC, efficiency change indexes; TC, technical change indexes.

#### 5.3.2 Temporal analysis of GML indexes and its decomposition

**Figure 4:**
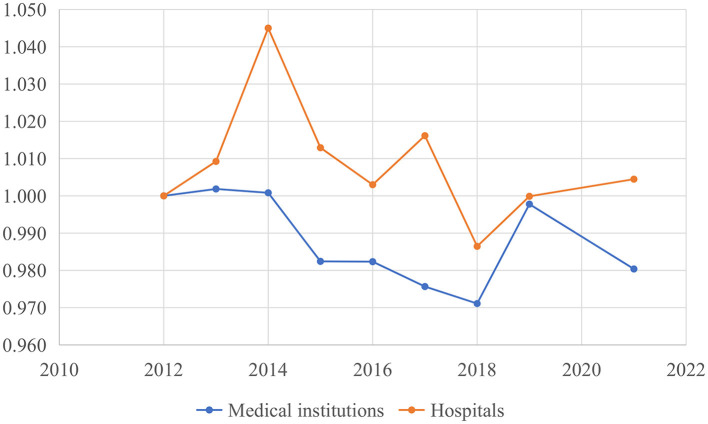
Cumulative growth of China's healthcare global malmquist-luenberger (GML) index.

Figure 4 illustrates the dynamic trajectory of cumulative healthcare TFP growth among medical institutions and hospitals. There was substantial volatility in the growth of TFP within China's mainland healthcare sector. TFP among medical institutions generally exhibited downward trend, aligning with the primary findings of efficiency values presented in Figure 1 and Table 4. However, there was a significant upswing in healthcare productivity during 2018–2019. This notable increase was primarily linked to technological progress, as indicated by the TC index at 1.03, signifying a key driver of healthcare productivity growth. The years 2014, 2017, and 2019 stood out as distinct peaks for hospitals, during which technological progress played a prominent role in driving healthcare productivity improvements. In contrast, improvements in technical efficiency were evident in 2013 and 2021 among both medical institutions and hospitals.

### 5.4 Hierarchy analysis

Table 7 illustrates healthcare efficiency among three subgroups within both medical institutions and hospitals, based on the proportion of primary services volume, primary medical staff, and total GDP. For medical institutions, efficiency among low-level group, middle-level group, and high-level group in term of primary service volume was 0.940, 0.960, and 0.965, respectively, while the figure for these three subgroups in term of primary medical staff was 0.940, 0.950, and 0.988, respectively. Similarly, the healthcare efficiency of GDP subgroups among medical institutions was 0.950, 0.951, and 0.974, respectively. The efficiency scores of hospitals were consistent with those of medical institutions. For hospital, efficiency among low-level group, middle-level group, and high-level group in term of primary service volume was 0.912, 0.931, and 0.949, respectively. Efficiency scores for three subgroups of primary service volume among hospitals stood at 0.912, 0.923, and 0.967, respectively. Efficiency scores for GDP subgroups among hospitals were 0.900, 0.932, and 0.960, respectively. These results suggest that regions with higher levels of primary service volume, primary medical staff, and GDP demonstrated correspondingly elevated level of healthcare efficiency. Notably, the GML indexes and their decomposed differences within each group did not exhibit any significant directional shifts (see Supplementary Table 10).

**Table 7:** Average healthcare efficiency scores by subgroups (2012–2021).

**Subgroups**	**Medical institutions**	**Hospitals**
**Primary services volume**		
Low-level group (Q1)	0.940	0.912
Middle-level group (Q2, Q3)	0.960	0.931
High-level group (Q4)	0.965	0.949
**Primary medical staff**		
Low-level group (Q1)	0.940	0.912
Middle-level group (Q2, Q3)	0.950	0.923
High-level group (Q4)	0.988	0.967
**GDP**		
Low-level group (Q1)	0.950	0.900
Middle-level group (Q2, Q3)	0.951	0.932
High-level group (Q4)	0.974	0.960

Average healthcare efficiency scores are sorted by three grouping criteria, namely the proportion of primary service volume, the proportion of primary medical staff, and the gross regional product (GDP).

### 5.5 Validity test and robustness checks

This study re-evaluated the efficiency scores of each DMU by incorporating the data of 2020. Additionally, we conducted a comparative analysis between the original efficiency values and those adjusted for variations in input or output indicators to validate the robustness of healthcare efficiency results. Table 8 presents a significant reduction in healthcare efficiency of both medical institutions and hospitals in 2020, leading to a decline in their average efficiency values. The GML indexes and TC indexes of hospitals were also affected and decreased. However, the technical efficiency of medical institutions increased by 1.4% in 2020 compared to 2019, resulting in an average year-by-year increase of 0.3% in the EC indexes (see Supplementary Table 11). Considering the ongoing influence of the COVID-19 epidemic in 2021, we excluded the 2021 data to perform a sensitivity analysis to examine the robustness of the results. Supplementary Table 12 present the healthcare efficiency scores of medical institutions and hospitals from 2012 to 2019, which were consistent with the main results of this study.

**Table 8:** Changes in average TFP and the decomposition of medical services in China before and after adding the 2020 data.

	**Before adding 2020**	**After adding 2020**
**Medical institutions**		
Efficiency scores	0.956	0.936
GML indexes	0.990	0.982
TC indexes	0.995	0.983
EC indexes	0.998	1.001
**Hospitals**		
Efficiency scores	0.930	0.911
GML indexes	1.002	0.993
TC indexes	1.009	1.002
EC indexes	0.994	0.993

GML = TC^*^EC.

GML, global Malmquist_luenberger indexes; EC, efficiency change indexes; TC, technical change indexes.

A commonly used method to validate DEA efficiency values is to add or remove certain input-output variables. In this study, the input indicator of total costs (*X*_5_) and the output indicator of total income (*Y*_4_) were excluded for medical institutions. Similarly, the input indicator of per-episode inpatient costs (*I*_5_) was removed for hospitals. Table 9 displays the spearman rank correlation coefficients for healthcare efficiency scores before and after incorporating the 2020 data and removing certain input-output indicators for both medical institutions and hospitals. The coefficients were as follows: 0.770 (*p* = 0.000) and 0.817 (*p* = 0.000) for medical institutions, as well as 0.834 (*p* = 0.000) and 0.836 (*p* = 0.000) for hospitals. These results indicate a strong positive correlation between efficiency values before and after adjustment for both medical institutions and hospitals. Furthermore, the Wilcoxon test showed that there was no significant difference in the efficiency values before and after adjustment. These dual test outcomes affirmed the robustness of healthcare efficiency values.

**Table 9:** Changes in the efficiency scores before and after adjustment.

	**Spearman rank correlation coefficient (β)**	**Mann-Whitney *U*-test (*Z*)**
**Medical institutions**		
Added 2020 data	0.770^***^	1.061
Deleted specific inputs and outputs	0.817^***^	2.428^*^
**Hospitals**		
Added 2020 data	0.834^***^	2.041^*^
Deleted specific inputs and outputs	0.836^***^	1.634

^*^p < 0.05,

^**^p < 0.01,

^***^p < 0.001.

The traditional DEA model and the Malmquist productivity index were employed to measure relative healthcare efficiency and productivity changes over the 2012–2021 period. In China's mainland, medical resources at medical institutions and hospitals are uniformly allocated by superior governments, making it difficult for DMUs to improve efficiency by adjusting medical inputs. Therefore, we applied an output-oriented DEA model. Additionally, the VRS model was used in this study because inputs and outputs of the 31 regions in China exhibit large differences in magnitude due to issues of imbalanced development. Supplementary Tables 13, 14 present the technical and scale efficiency of medical institutions and hospitals. The average score of technical efficiency, pure technical efficiency and scale efficiency in medical institutions was 0.993, 0.996, and 0.997, respectively, while in hospitals, during 2012–2021, they were 0.949, 0.991, and 0.957, respectively. The Malmquist index summary of annual geometric means from 2012 to 2021 in medical institutions and hospitals is shown in Supplementary Tables 15, 16. On average, total factor productivity of medical institutions decreased by 1.7%, with technical change decreasing by 1.6% and technical efficiency change decreasing slightly by 0.1%. During 2012–2021, total factor productivity and technical change of hospitals decreased by 4 and 3.9%, respectively. Technical change was the main contributor to healthcare productivity, which was consistent with the conclusion of this study. Notably, the average pure technical efficiency of 0.998 was less than the average scale efficiency of 1.002 in hospitals, indicating an inefficient use of medical inputs.

## 6 Discussion

This study utilized the SBM-DDF model within three-stage DEA analysis framework to quantify healthcare efficiency in China. We employed regional balanced panel data from 2012 to 2021, incorporating undesirable outputs and medical quality indicators into input-output variables. The GML index was used to examine changes in China's mainland healthcare efficiency and its components. The analysis also decomposed the sources of healthcare inefficiency. Additionally, we categorized different regions into low, middle, and high subgroups using three grouping standards: the proportion of primary service volume, the proportion of primary medical staff, and total GDP. The quartile method was employed to compare the efficiency scores and GML indexes of each group. We found that China's overall healthcare efficiency experienced fluctuations between 2012 and 2021. Notably, the average efficiency value of medical institutions (0.956) was slightly higher than that of hospitals (0.930). In line with the evaluation of healthcare efficiency across various regions, regions with higher healthcare efficiency predominantly resided in eastern China.

The healthcare TFP in China experienced an average decrease of 1% from 2012 to 2021, while the TC indexes and EC indexes showed an average degradation of 0.5 and 0.2%, respectively. In contrast, hospital TFP and TC indexes increased by 2 and 9%, respectively. The opposite trends in TFP between medical institutions and hospitals may be attributed to the fact that medical institutions include both primary and professional medical institutions, with lower levels of service and technological development compared to hospitals. Regions experiencing an increase in TFP were primarily influenced by the growth of TC, indicating that medical technology progress played a pivotal role in enhancing healthcare efficiency within China's mainland. Additionally, the input inefficiency of China's medical services was attributed to an excess in institutional proliferation, while desirable output inefficiency arose due to a scarcity of outpatient visits and inpatient surgery volume. Inefficiency related to undesirable outputs in medical institutions and hospitals could be traced to fluctuations in mortality rates and LOS, respectively.

It is noteworthy that from 2012 to 2021, an increase in the proportion of primary services, primary medical staff, and total GDP was associated with heightened healthcare efficiency among both medical institutions and hospitals. This underscores the importance of prioritizing primary medical services in China. Regions that prioritized higher levels of primary healthcare delivery and had a more advanced economic status invariably exhibited superior healthcare efficiency. This outcome conveys the pivotal significance of judicious medical resources allocation, strategic distribution of premium medical resources into primary settings, and the augmentation of medical proficiency within primary medical institutions. Collectively, these efforts contribute to a comprehensive enhancement in the efficiency of regional healthcare systems.

Despite the growing literature on the efficiency of Chinese healthcare system, less attention has been given to examine the undesirable outputs linked to healthcare services. Most studies took labor-capital volumes and staff-oriented medical activities as inputs and outputs, respectively. The existing results showed that healthcare efficiency in China generally had fluctuating upward trends (11, 12) with significant regional differences based on the traditional DEA model. However, Yu et al. found that healthcare TFP in China continued to decline slowly in 2009–2015, which was consistent with the conclusion of this study (69). They employed SBM model and GML indexes by including environmental pollution resulting from the incineration of medical waste as an undesirable output. In contrast, this study used in-hospital mortality rate and LOS, which were directly relevant to the production process of medical services (11). And the utilization of SBM-DDF model in this study, compared with the DEA model, enables the mixed application of absolute data and relative data, including mortality rates and bed occupancy rates.

Although medical institutions and hospitals play a critical role in ensuring the delivery of medical services, less is known about how to improve the efficiency and quality of healthcare provided (64, 70). The inefficiency analysis of this study provided insight into the input-output slacks of medical service in China. We found that the redundant number and costs of medical sectors, insufficient outpatient visits and surgery volumes, and the slacks of mortality rates and LOS were the main reasons for healthcare inefficiency among medical institutions and hospitals. To improve the efficiency scores, policymakers should first implement adequate supervision measures to control medical costs and regulate undesirable healthcare provider behavior. The overuse of the number of medical services provided may encourage healthcare providers to gain better performance and increase efficiency scores at the expense of quality, adversely affecting health outcomes and promotion. Therefore, it is necessary to incorporate medical quality in performance evaluation. The findings of this study also offer certain evidence for the benefit of promoting primary care, including primary services volume and primary medical staff, and GDP. Policymakers should place more emphasis on equalizing high-quality primary medical services and hierarchic healthcare in China by offer sufficient subsidies to primary institutions. Additionally, considering the regional difference of healthcare efficiency that has been widely recognized, it is crucial to strengthen regional health planning and balance the development of regional healthcare. It is necessary to decrease medical technology gap across eastern district, western district and central district.

The study has several limitations that should be taken into account when interpreting the results. Firstly, it did not consider the impact of inter-regional medical treatment on regional healthcare efficiency. Secondly, it lacked exploration of the relationship between healthcare efficiency and quality in depth. Thirdly, the research period of this paper was relatively short due to data limitation. Additionally, regional data from official yearbooks was self-reported by single province, which might cause reporting inconsistencies. More sensitivity analyses could be conducted to verify reported outcomes. Lastly, the selection of input-output indicators was somewhat subjective and lacks of normative conceptual framework. Despite these limitations, the current findings hold important implications for healthcare policymaking in China.

## 7 Conclusions

We utilized a three-stage DEA method with the SBM-DDF model to analyze the efficiency performance of medical institutions and hospitals, employing the GML index to identify temporary changes in efficiency across 31 regions in China's mainland. We found that the healthcare TFP among medical institutions experienced an average decrease of 1% from 2012 to 2021, while hospital TFP increased by 2%. Medical technology emerged as the primary driver of efficiency in medical service across regions. The healthcare inefficiency was primarily attributed to the proliferation of institutions and insufficient medical service volumes. Additionally, regions prioritizing primary medical services and boasting higher GDP levels exhibited superior healthcare efficiency. These findings are expected to inform policymakers' efforts in building a value-based and efficient health service system.

## Data availability statement

Publicly available datasets were analyzed in this study. This data can be found at: http://www.nhc.gov.cn/mohwsbwstjxxzx/tjtjnj/tjsj_list.shtml; http://www.nhc.gov.cn/mohwsbwstjxxzx/tjtjnj/202305/6ef68aac6bd14c1eb9375e01a0faa1fb.shtml; http://cnki.nbsti.net/CSYDMirror/area/Yearbook/Single/N2022040097?z=D26.

## Author contributions

BF: Writing – original draft, Writing – review & editing, Conceptualization. ML: Writing – review & editing.

## Footnotes

^1^Regions refer to DMUs for ease of understanding.

^2^If the likelihood ratio test conducted on the SFA model rejects the null hypothesis signifying the absence of an inefficiency term, the subsequent recourse to direct Tobit regression assumes precedence. It is worth noting, however, that while Tobit regression is engaged in this context, its efficacy in delineating statistical noise remains limited.

^3^Undesirable outputs are adjusted with reference to the input adjustments method.

^4^It is worth noting that regions included in the low-level primary medical service volume group and the low-level primary medical staff group are exactly the same, namely Beijing, Heilongjiang, Liaoning, Ningxia, Qinghai, Shanghai, Tianjin, and Xinjiang.

^5^Life expectancy in each region should theoretically be included in the output variables as this indicator directly reflects the output effect of healthcare economic system. Unfortunately, a comprehensive dataset detailing year-to-year life expectancy across regions in China is not available. Currently, only census data from the years 2000, 2010, and 2020 is accessible.

## References

[1] National Health Commission of the People's Republic of China. China Health Statistical Yearbook. (2011). Available online at: http://www.nhc.gov.cn/mohwsbwstjxxzx/tjtjnj/tjsj_list.shtml (accessed November 17, 2022).

[2] Yu B Wang T He C Zheng Guo S. Research on the efficiency of China health service system based on three-stage DEA. Manag Rev. (2023) 34:312. 10.14120/j.cnki.cn11-5057/f.20210616.007

[3] Wang Q Wei J. Competition, insurance and efficiency of hospital market: based on the two stage analysis of DEA model. Econ Probl. (2013) 4:17–21. 10.16011/j.cnki.jjwt.2013.04.001

[4] Afzali HHA Moss JR Mahmood MA. A conceptual framework for selecting the most appropriate variables for measuring hospital efficiency with a focus on Iranian public hospitals. Health Serv Manage Res. (2009) 22:81–91. 10.1258/hsmr.2008.008020 19401501

[5] Shi Y Xie Y Chen H Zou W. Spatial and temporal differences in the health expenditure efficiency of China: reflections based on the background of the COVID-19 pandemic. Front Public Health. (2022) 10:879698. 10.3389/fpubh.2022.879698 35493397 PMC9051031

[6] Wang X Sun X Gong F Huang Y Chen L Zhang Y. The Luohu model: a template for integrated urban healthcare systems in China. Int J Integr Care. (2018) 18:3. 10.5334/ijic.3955 30483036 PMC6199563

[7] Ng YC. The productive efficiency of the health care sector of China. RRS. (2008) 38:381–93. 10.52324/001c.827236153787

[8] Guo B Zhang J Fu X. Evaluation of unified healthcare efficiency in China: a meta-frontier non-radial directional distance function analysis during 2009-2019. Front Public Health. (2022) 10:876449. 10.3389/fpubh.2022.876449 35669743 PMC9163441

[9] Ye Z Jiang Y. The impact of a pilot integrated care model on the quality and costs of inpatient care among Chinese elderly: a difference-in-difference analysis of repeated cross-sectional data. Cost Eff Resour Alloc. (2022) 20:28. 10.1186/s12962-022-00361-4 35752860 PMC9233857

[10] Cylus J Papanicolas I Smith PC. Using data envelopment analysis to address the challenges of comparing health system efficiency. Glob Pol. (2017) 8:60–8. 10.1111/1758-5899.1221223891192

[11] Yang Y Zhang L Zhang X Yang M Zou W. Efficiency measurement and spatial spillover effect of provincial health systems in China: based on the two-stage network DEA model. Front Public Health. (2022) 10:952975. 10.3389/fpubh.2022.952975 36262222 PMC9574077

[12] Deng J Deng W Yang Z Huang L. Analysis on the hospital medical service efficiency in China based on Malmquist Index. Chin Health Econ. (2024) 2024:1–8.

[13] Xia F Leng Y Zhang R Du X. Analysis on the change of service efficiency of primary medical institutions before and after medical reform in China. Health Econ Res. (2018) 2:41–5. 10.14055/j.cnki.33-1056/f.20180206.009

[14] Liu Z Zhang X Yang D. Research on efficiency change of Chinese government health investment: based on panel three stage DEA model. J Central Univ Fin Econ. (2014) 6:97–104.

[15] Karagiannis G. On structural and average technical efficiency. J Prod Anal. (2015) 43:259–67. 10.1007/s11123-015-0439-x

[16] Hossain MdK Kamil AA Baten MdA Mustafa A. Stochastic frontier approach and data envelopment analysis to total factor productivity and efficiency measurement of bangladeshi rice. PLoS ONE. (2012) 7:e46081. 10.1371/journal.pone.0046081 23077500 PMC3471888

[17] Wen F Fang X Shan A Khanal R Huang J. How is the medical service efficiency in China? An empirical analysis using stochastic frontier approach and gravity models. Int J Health Plan Manag. (2022) 37:2949–63. 10.1002/hpm.3534 35775602

[18] Jacobs R Smith PC Street A. Measuring Efficiency in Health Care: Analytic Techniques and Health Policy. 1st ed. Cambridge: Cambridge University Press (2006). Available online at: https://www.cambridge.org/core/product/identifier/9780511617492/type/book (accessed March 19, 2024).

[19] Charnes A Cooper WW Rhodes E. Measuring the efficiency of decision making units. Eur J Oper Res. (1978) 2:429–44. 10.1016/0377-2217(78)90138-8

[20] Banker RD Charnes A Cooper WW. Some models for estimating technical and scale inefficiencies in data envelopment analysis. Manage Sci. (1984) 30:1078–92. 10.1287/mnsc.30.9.107819642375

[21] Fukuyama H Weber WL. A directional slacks-based measure of technical inefficiency. Socioecon Plann Sci. (2009) 43:274–87. 10.1016/j.seps.2008.12.001

[22] Nunamaker TR. Efficiency Measurement and Medicare Reimbursement in Nonprofit Hospitals: an Investigation of the Usefulness of Data Envelopment Analysis. University Microfilms International (1983). Available online at: https://ci.nii.ac.jp/ncid/BB01327743 (accessed September 1, 2023).

[23] Sherman HD. Hospital efficiency measurement and evaluation. Empirical test of a new technique. Med Care. (1984) 22:922–38. 10.1097/00005650-198410000-00005 6436590

[24] Panwar A Olfati M Pant M Snasel V. A review on the 40 years of existence of data envelopment analysis models: historic development and current trends. Arch Computat Methods Eng. (2022) 29:5397–426. 10.1007/s11831-022-09770-3 35702633 PMC9184254

[25] Charnes A Cooper WW Golany B Seiford L Stutz J. Foundations of data envelopment analysis for Pareto-Koopmans efficient empirical production functions. J Econom. (1985) 30:91–107. 10.1016/0304-4076(85)90133-2

[26] Kohl S Schoenfelder J Fügener A Brunner JO. The use of Data Envelopment Analysis (DEA) in healthcare with a focus on hospitals. Health Care Manag Sci. (2019) 22:245–86. 10.1007/s10729-018-9436-8 29478088

[27] Tone K. A slacks-based measure of efficiency in data envelopment analysis. Eur J Operat Res. (2001) 2001:5. 10.1016/S0377-2217(99)00407-5

[28] Tone K Tsutsui M. Dynamic DEA with network structure: a slacks-based measure approach. Omega. (2014) 42:124–31. 10.1016/j.omega.2013.04.002

[29] Pastor JT Aparicio J Zofío JL. Shephard's Input and Output Distance Functions: Cost and Revenue Efficiency Decompositions. SpringerLink (2019). Available online at: https://link.springer.com/chapter/10.1007/978-3-030-84397-7_3 (accessed March 19, 2024).

[30] Luenberger DG. New optimality principles for economic efficiency and equilibrium. J Optim Theory Appl. (1992) 75:221–64. 10.1007/BF00941466

[31] Re RF. Theory and application of directional distance functions. J Product Anal. (2000) 13:93–103. 10.1023/A:1007844628920

[32] Cai N Cong Y Li Z. Technology innovation and China's industry energy saving and emission reduction efficiency: analysis on regional difference based on SBM-DDF and panel data model. Econ Theor Bus Manag. (2014) 6:57–70.

[33] Malmquist S. Index numbers and indifference surfaces. Trabajos de Estadistica. (1953) 4:209–42. 10.1007/BF03006863

[34] Caves DW Christensen LR Diewert WE. The economic theory of index numbers and the measurement of input, output, and productivity. Econometrica. (1982) 50:1393–414. 10.2307/1913388

[35] Bjurek H Førsund FR Hjalmarsson L. Malmquist productivity indexes: an empirical comparison. In:Färe R Grosskopf S Russell RR, editors. Index Numbers: Essays in Honour of Sten Malmquist. Dordrecht: Springer Netherlands (1998). p. 217–39.

[36] Ray S Desli E. Productivity growth, technical progress, and efficiency change in industrialized countries: comment. Am Econ Rev. (1997) 87:1033–9.

[37] Lall P Featherstone AM Norman DW. Productivity growth in the western hemisphere (1978-94): the Caribbean in perspective. J Product Anal. (2002) 17:213–31. 10.1023/A:1015008020851

[38] Chung Y Fare R. Productivity and undesirable outputs: a directional distance function approach. Microeconomics. (1997) 51:229–40. 10.1006/jema.1997.0146

[39] Pastor JT Lovell CAK. A global Malmquist productivity index. Econ Lett. (2005) 88:266–71. 10.1016/j.econlet.2005.02.013

[40] O'Neill L Rauner M Heidenberger K Kraus M. A cross-national comparison and taxonomy of DEA-based hospital efficiency studies. Socioecon Plann Sci. (2008) 42:158–89. 10.1016/j.seps.2007.03.001

[41] Fried HO Lovell CAK Schmidt SS Yaisawarng S. Accounting for environmental effects and statistical noise in data envelopment analysis. J Product Anal. (2002) 17:157–74. 10.1023/A:1013548723393

[42] Luo D. A note on estimating managerial inefficiency of three-stage DEA model. Statist Res. (2012) 29:104–7. 10.19343/j.cnki.11-1302/c.2012.04.017

[43] Deng Z Wu C Feng Y Wang J. Analysis on the regional differences in the efficiency of public service supply in China. Econ Geogr. (2014) 34:28–33. 10.15957/j.cnki.jjdl.2014.05.007

[44] Du T. Dynamic Evaluation and Promotion of Healthcare Service Efficiency Considering Quality and Equity. Beijing: Beijing Institute of Technology (2021). Available online at: https://kns.cnki.net/kcms2/article/abstract?v=Vof-4b7nxdAGbXBMN4RfDauVBH2mo30IH-AdHiaMQyOW2qVHQSvhRzLZ-JxcDr1cNLZT_G362gu_D5XxuEeNd1Eyd8OMvFuUtkHnmW5D9W0-rhnszUFpwNc3bRfaO7KrMQ-VkcVI3FdauhrcFsbyww==&uniplatform=NZKPT&language=CHS

[45] Pang R. Evaluation of Chinese hospital's operation performances-two stages analysis based on data envelopment analysis (DEA). Nan Kai Econ Stud. (2006) 4:71–81.

[46] Wang W Pan J. Analysis on the efficiency of 14 division-level hospitals of Xinjiang production and construction corps based on DEA model. Chin Health Econ. (2013) 32:78–80. 10.6106/kJCEM.2013.14.3.078

[47] Yang F Fu C Yao Y Mao Z. Analysis of the technical efficiency and total factor productivity of medical and health resources in the county of Hubei Province. Chin Health Resour. (2017) 20:60–4. 10.13688/j.cnki.chr.2017.16339

[48] Zhang X Liu Z. An analysis on hospital efficiency at provincial level and its influencing factors in China-DEA-Tobit estimation based on the provincial panel data. East China Econ Manag. (2014) 28:172–6.27490260

[49] Shen S Zheng Q. Research on health production efficiency and its influencing factors in China. J Sun Yat-sen Univ. (2017) 57:153. 10.13471/j.cnki.jsysusse.2017.06.016

[50] Oh D. A global Malmquist-Luenberger productivity index. J Prod Anal. (2010) 34:183–97. 10.1007/s11123-010-0178-y

[51] Nayar P Ozcan YA Yu F Nguyen AT. Benchmarking urban acute care hospitals: efficiency and quality perspectives. Health Care Manage Rev. (2013) 38:137. 10.1097/HMR.0b013e3182527a4c 22469911

[52] Bilsel M Davutyan N. Hospital efficiency with risk adjusted mortality as undesirable output: the Turkish case. Ann Oper Res. (2014) 221:73–88. 10.1007/s10479-011-0951-y

[53] Yang F Wei F Li Y Huang Y Chen Y. Expected efficiency based on directional distance function in data envelopment analysis. Comput Industr Eng. (2018) 125:33–45. 10.1016/j.cie.2018.08.010

[54] Liu Z Xin L. The impact of the “Belt and Road” construction on green total factor productivity in China's key provinces along the route. China Popul Resour Environ. (2018) 28:87–97.

[55] Wang B Wu Y Yan P. Environmental efficiency and environmental total factor productivity growth in China's regional economies. Econ Res J. (2010) 45:95–109.

[56] Liu R An T. Trend and factor analysis of Chinese economic growth performance under restrictions of resource and environment—a research based on a new method of productivity index's construction and decomposition. Econ Res J. (2012) 47:34–47.

[57] Coelli T. A Guide to Frontier version 4. 1: A Computer Program for Stochastic Frontier Production and Cost Fu. (1996). Available online at: https://www.semanticscholar.org/paper/A-Guide-to-Frontier-version-4.-1%3A-A-Computer-for-Fu-Coelli/3e54aec64c45c8e9b95665dc312f78e7ce3296cc (accessed March 30, 2024).

[58] National Bureau of Statistic. China Statistical Yearbook. (1999). Available online at: http://www.stats.gov.cn/tjsj./ndsj/ (accessed November 17, 2022).

[59] CNKI. China Population and Employment Statistical Yearbook. (2023). Available online at: http://cnki.nbsti.net/CSYDMirror/area/Yearbook/Single/N2022040097?z=D26 (accessed September 3, 2023).

[60] Retzlaff-Roberts D Chang CF Rubin RM. Technical efficiency in the use of health care resources: a comparison of OECD countries. Health Policy. (2004) 69:55–72. 10.1016/j.healthpol.2003.12.002 15484607

[61] Berger M Messer J. Public financing of health expenditures, insurance, and health outcomes. Appl Econ. (2002) 34:2105–13. 10.1080/00036840210135665

[62] Hollingsworth B Wildman J. The efficiency of health production: re-estimating the WHO panel data using parametric and non-parametric approaches to provide additional information. Health Econ. (2003) 12:493–504. 10.1002/hec.751 12759918

[63] Thornton D. Constructing and testing a framework for dynamic risk assessment. Sex Abuse. (2002) 14:139–53. 10.1177/107906320201400205 11961888

[64] Preker AS Harding A. Innovations in Health Service Delivery: the Corporatization of Public Hospitals. World Bank (2016). Available online at: https://documents.worldbank.org/en/publication/documents-reports/documentdetail/313081515574955479/Innovations-in-health-service-delivery-the-corporatization-of-public-hospitals (accessed March 25, 2024).

[65] Dharmapala PS. Adding value in healthcare service by improving operational efficiency using Data Envelopment Analysis. Int J Operat Res. (2009) 2009:24530. 10.1504/IJOR.2009.02453035009967

[66] Girginer N Köse T Uçkun N. Efficiency analysis of surgical services by combined use of data envelopment analysis and gray relational analysis. J Med Syst. (2015) 39:56. 10.1007/s10916-015-0238-y 25764507

[67] Simar L Wilson PW. Estimation and inference in two-stage, semi-parametric models of production processes. J Econom. (2007) 136:31–64. 10.1016/j.jeconom.2005.07.009

[68] Chen W Zhang L Ma T Liu Q. Research on three stage DEA model. Syst Eng. (2014) 32:144–9.

[69] Yu J Liu Z Zhang T Hatab AA Lan J. Measuring productivity of healthcare services under environmental constraints: evidence from China. BMC Health Serv Res. (2020) 20:673. 10.1186/s12913-020-05496-9 32698810 PMC7374832

[70] Wang M Fang H Tao H Cheng Z Lin X Cai M. Bootstrapping data envelopment analysis of efficiency and productivity of county public hospitals in Eastern, Central, and Western China after the public hospital reform. Curr Med Sci. (2017) 37:681–92. 10.1007/s11596-017-1789-6 29058280

