# Supplementary material for: Evaluation of healthcare efficiency in China: a three-stage data envelopment analysis of directional slacks-based measure
Source: Front Public Health. 2024 May 30;12:1393143. doi: 10.3389/fpubh.2024.1393143 (PMC11169848; doi:10.3389/fpubh.2024.1393143)
Supplement: Supplementary file 3 [file Data_Sheet_3.docx]

**Appendix A: Table S1-S16**

Table S1. Inputs and outputs indicators of healthcare efficiency among hospitals.

| Variable categories | | Variables |
| --- | --- | --- |
| Inputs | Medical facilities | Number of hospitals ($I_{1})$  Number of beds ($I_{2}$) |
|  | Medical personnel | Number of doctors ($I_{3}$)  Number of registered nurses ($I_{4}$) |
|  | Medical expenditure | Per-episode inpatient costs ($I_{5}$) |
| Outputs | Outpatient services | Number of outpatient visits ($O_{1}$) |
|  | Inpatient services | Number of hospitalizations ($O_{2}$)  Inpatient surgery volumes ($O_{3}$)  Occupancy rate of hospital beds ($O_{4}$) |
| Undesirable outputs | Inpatient quality | LOS ($O_{5}$) |

Abbreviations: LOS, length of stay.

Table S2. Descriptive statistics of input-output variables among hospitals.

| Categories | Variables | Sample size | Mean | Standard deviation | Minimum | Maximum |
| --- | --- | --- | --- | --- | --- | --- |
| Inputs | $I_{1}$ | 310 | 0.243 | 0.083 | 0.100 | 0.489 |
|  | $I_{2}$ | 310 | 14.180 | 4.063 | 7.432 | 32.890 |
|  | $I_{3}$ | 310 | 19.960 | 5.798 | 4.003 | 42.780 |
|  | $I_{4}$ | 310 | 43.250 | 9.858 | 17.100 | 70.790 |
|  | $I_{5}$ | 310 | 3.244 | 3.916 | 0.531 | 22.010 |
| Desirable outputs | $O_{1}$ | 310 | 244.700 | 125.400 | 96.570 | 713.100 |
|  | $O_{2}$ | 310 | 1239.000 | 262.900 | 355.100 | 1915.000 |
|  | $O_{3}$ | 310 | 363.300 | 159.700 | 71.390 | 1578.000 |
|  | $O_{4}$ | 310 | 82.140 | 8.438 | 48.300 | 99.300 |
| Undesirable outputs | $O_{5}$ | 310 | 9.530 | 0.809 | 7.700 | 12.800 |

Table S3-1. Average growth rate of various inputs and outputs among medical institutions from 2012 to 2021.

| Year | Input variables | | | | | Desirable output variables | | | | Undesirable output variable |
| --- | --- | --- | --- | --- | --- | --- | --- | --- | --- | --- |
|  | $X_{1}$ | $X_{2}$ | $X_{3}$ | $X_{4}$ | $X_{5}$ | $Y_{1}$ | $Y_{2}$ | $Y_{3}$ | $Y_{4}$ | $Y_{5}$ |
| 2013 | 2.536% | 6.830% | 11.476% | 7.985% | 14.729% | 6.180% | 7.172% | 7.926% | 14.147% | 6.814% |
| 2014 | 0.722% | 3.498% | 7.942% | 6.783% | 12.053% | 3.936% | 6.516% | 10.047% | 12.299% | 5.220% |
| 2015 | 0.214% | 5.069% | 7.900% | 6.272% | 9.640% | 1.204% | 2.894% | 3.942% | 8.522% | 3.150% |
| 2016 | -0.014% | 4.997% | 8.197% | 5.634% | 8.234% | 3.097% | 7.867% | 11.557% | 8.198% | 0.763% |
| 2017 | 0.331% | 6.237% | 8.464% | 7.149% | 5.822% | 3.170% | 7.574% | 10.104% | 5.234% | -0.758% |
| 2018 | 1.093% | 6.405% | 7.745% | 5.841% | 7.519% | 1.526% | 4.396% | 10.291% | 6.896% | 0.000% |
| 2019 | 1.017% | 7.201% | 8.452% | 4.794% | 7.322% | 4.957% | 4.404% | 12.296% | 10.016% | -2.290% |
| 2020 | 1.523% | 5.658% | 5.932% | 3.335% | 11.356% | -11.225% | -13.290% | -3.848% | 3.085% | 19.297% |
| 2021 | 0.783% | 4.942% | 6.599% | 3.839% | 3.060% | 9.443% | 7.230% | 21.600% | 12.142% | -5.632% |
| Mean | 0.912% | 5.649% | 8.078% | 5.737% | 8.859% | 2.476% | 3.863% | 9.324% | 8.949% | 2.952% |

Table S3-2. Average growth rates of inputs and outputs among hospital from 2012 to 2021.

| Year | Input variables | | | | | Desirable output variables | | | | Undesirable output variable |
| --- | --- | --- | --- | --- | --- | --- | --- | --- | --- | --- |
|  | $I_{1}$ | $I_{2}$ | $I_{3}$ | $I_{4}$ | $I_{5}$ | $O_{1}$ | $O_{2}$ | $O_{3}$ | $O_{4}$ | $O_{5}$ |
| 2013 | 6.642% | 7.080% | 11.538% | 10.023% | 5.970% | 7.875% | 9.623% | 8.468% | -1.403% | -2.334% |
| 2014 | 4.658% | 5.402% | 8.863% | 8.355% | 2.526% | 8.399% | 9.983% | 10.158% | -1.316% | -2.519% |
| 2015 | 6.678% | 6.840% | 8.340% | 7.446% | 3.338% | 3.754% | 4.536% | 4.630% | -3.129% | -0.729% |
| 2016 | 5.629% | 6.539% | 8.545% | 6.722% | 0.636% | 6.029% | 8.859% | 11.348% | -0.169% | -2.336% |
| 2017 | 6.575% | 7.157% | 8.000% | 7.587% | -3.356% | 5.180% | 7.974% | 10.491% | -0.728% | -1.708% |
| 2018 | 6.289% | 6.261% | 7.028% | 6.523% | 0.552% | 4.026% | 6.031% | 10.710% | -0.761% | -0.452% |
| 2019 | 4.075% | 5.879% | 7.189% | 5.319% | 3.122% | 7.408% | 5.763% | 12.396% | -0.633% | -1.117% |
| 2020 | 3.027% | 4.981% | 4.647% | 3.854% | 6.372% | -13.521% | -13.145% | -3.979% | -14.272% | 3.072% |
| 2021 | 3.323% | 5.003% | 5.852% | 3.969% | 2.243% | 16.881% | 9.458% | 19.752% | 3.126% | -2.706% |
| Mean | 5.211% | 6.127% | 7.778% | 6.644% | 2.378% | 5.115% | 5.454% | 9.330% | -2.143% | -1.203% |

Table S4. The average healthcare efficiency in various regions and their ranking.

| Regions |  | Medical institutions | | |  | Hospitals | | | | |
| --- | --- | --- | --- | --- | --- | --- | --- | --- | --- | --- |
|  |  | Sort by efficiency score |  | Average efficiency score |  | Sort by efficiency score |  | | Average efficiency score | |
| Fujian |  | 1 |  | 1.000 |  | 16 | |  | | 0.951 |
| Jiangsu |  | 2 |  | 0.998 |  | 19 | |  | | 0.933 |
| Shanghai |  | 3 |  | 0.998 |  | 8 | |  | | 0.991 |
| Yunnan |  | 4 |  | 0.997 |  | 1 | |  | | 1.000 |
| Jiangxi |  | 5 |  | 0.996 |  | 13 | |  | | 0.960 |
| Hunan |  | 6 |  | 0.994 |  | 10 | |  | | 0.974 |
| Zhejiang |  | 7 |  | 0.994 |  | 4 | |  | | 0.995 |
| Anhui |  | 8 |  | 0.993 |  | 12 | |  | | 0.964 |
| Guangdong |  | 9 |  | 0.992 |  | 7 | |  | | 0.992 |
| Guizhou |  | 10 |  | 0.986 |  | 6 | |  | | 0.992 |
| Chongqing |  | 11 |  | 0.986 |  | 17 | |  | | 0.950 |
| Beijing |  | 12 |  | 0.983 |  | 14 | |  | | 0.957 |
| Tibet |  | 13 |  | 0.982 |  | 27 | |  | | 0.843 |
| Tianjin |  | 14 |  | 0.978 |  | 21 | |  | | 0.925 |
| Ningxia |  | 15 |  | 0.977 |  | 24 | |  | | 0.881 |
| Henan |  | 16 |  | 0.977 |  | 18 | |  | | 0.933 |
| Gansu |  | 17 |  | 0.975 |  | 9 | |  | | 0.986 |
| Guangxi |  | 18 |  | 0.974 |  | 2 | |  | | 1.000 |
| Hubei |  | 19 |  | 0.972 |  | 3 | |  | | 0.998 |
| Sichuan |  | 20 |  | 0.967 |  | 11 | |  | | 0.964 |
| Hainan |  | 21 |  | 0.953 |  | 28 | |  | | 0.833 |
| Xinjiang |  | 22 |  | 0.951 |  | 5 | |  | | 0.992 |
| Shanxi |  | 23 |  | 0.942 |  | 15 | |  | | 0.955 |
| Hebei |  | 24 |  | 0.935 |  | 20 | |  | | 0.928 |
| Qinghai |  | 25 |  | 0.919 |  | 30 | |  | | 0.815 |
| Shandong |  | 26 |  | 0.915 |  | 22 | |  | | 0.908 |
| Shanxi |  | 27 |  | 0.881 |  | 31 | |  | | 0.799 |
| Heilongjiang |  | 28 |  | 0.858 |  | 26 | |  | | 0.853 |
| Liaoning |  | 29 |  | 0.857 |  | 23 | |  | | 0.886 |
| Jilin |  | 30 |  | 0.854 |  | 25 | |  | | 0.858 |
| Inner Mongolia |  | 31 |  | 0.852 |  | 29 | |  | | 0.820 |

Table S5. The input-output inefficiency scores among hospitals between 2012 and 2021.

| Year | Input variables | | | | | Desirable output variables | | | | Undesirable output variable |
| --- | --- | --- | --- | --- | --- | --- | --- | --- | --- | --- |
|  | $I_{1}$ | $I_{2}$ | $I_{3}$ | $I_{4}$ | $I_{5}$ | $O_{1}$ | $O_{2}$ | $O_{3}$ | $O_{4}$ | $O_{5}$ |
| 2012 | 0.013 | 0.006 | 0.001 | 0.005 | 0.015 | 0.026 | 0.002 | 0.015 | 0.001 | 0.005 |
| 2013 | 0.011 | 0.005 | 0.000 | 0.005 | 0.012 | 0.021 | 0.002 | 0.016 | 0.002 | 0.003 |
| 2014 | 0.009 | 0.002 | 0.000 | 0.004 | 0.007 | 0.012 | 0.000 | 0.005 | 0.001 | 0.002 |
| 2015 | 0.012 | 0.004 | 0.000 | 0.005 | 0.007 | 0.024 | 0.001 | 0.015 | 0.005 | 0.002 |
| 2016 | 0.017 | 0.004 | 0.000 | 0.005 | 0.010 | 0.023 | 0.001 | 0.024 | 0.004 | 0.000 |
| 2017 | 0.014 | 0.005 | 0.000 | 0.004 | 0.007 | 0.018 | 0.001 | 0.020 | 0.004 | 0.001 |
| 2018 | 0.018 | 0.005 | 0.001 | 0.005 | 0.009 | 0.031 | 0.002 | 0.032 | 0.003 | 0.000 |
| 2019 | 0.016 | 0.004 | 0.001 | 0.006 | 0.010 | 0.025 | 0.001 | 0.025 | 0.005 | 0.000 |
| 2020 | 0.018 | 0.006 | 0.004 | 0.010 | 0.005 | 0.022 | 0.002 | 0.015 | 0.009 | 0.001 |
| 2021 | 0.014 | 0.005 | 0.001 | 0.005 | 0.009 | 0.022 | 0.001 | 0.019 | 0.004 | 0.002 |
| Mean | 0.014 | 0.005 | 0.001 | 0.005 | 0.009 | 0.022 | 0.001 | 0.019 | 0.004 | 0.002 |

Table S6-1. The input slacks and output slacks among medical institutions between 2012 and 2021.

| Year | $X_{1}$ | $X_{2}$ | $X_{3}$ | $X_{4}$ | $X_{5}$ | $Y_{1}$ | $Y_{2}$ | $Y_{3}$ | $Y_{4}$ | $Y_{5}$ |
| --- | --- | --- | --- | --- | --- | --- | --- | --- | --- | --- |
| 2012 | 5.261 | 10.768 | 3.570 | 12.840 | 28.177 | 218.245 | 538.810 | 130.193 | 46.955 | 0.060 |
| 2013 | 6.637 | 9.400 | 1.829 | 10.197 | 5.328 | 264.838 | 487.305 | 123.806 | 2.246 | 0.060 |
| 2014 | 8.896 | 6.456 | 2.588 | 13.875 | 8.433 | 289.307 | 270.147 | 142.611 | 50.212 | 0.066 |
| 2015 | 11.451 | 8.840 | 3.506 | 28.921 | 36.525 | 459.121 | 441.432 | 337.019 | 35.839 | 0.090 |
| 2016 | 12.268 | 7.455 | 3.976 | 28.175 | 75.519 | 519.197 | 399.190 | 358.096 | 65.244 | 0.094 |
| 2017 | 12.652 | 10.409 | 6.803 | 30.647 | 145.401 | 633.131 | 400.268 | 382.360 | 346.495 | 0.101 |
| 2018 | 13.824 | 11.437 | 5.449 | 32.327 | 554.838 | 705.053 | 491.171 | 426.824 | 762.262 | 0.107 |
| 2019 | 9.716 | 13.883 | 2.963 | 19.057 | 2.019 | 386.057 | 412.861 | 169.719 | 40.551 | 0.092 |
| 2021 | 10.371 | 21.003 | 10.541 | 28.785 | 42.181 | 877.026 | 865.644 | 207.038 | 33.351 | 0.125 |
| Mean | 10.119 | 11.072 | 4.581 | 22.758 | 99.825 | 483.553 | 478.536 | 253.074 | 153.684 | 0.088 |

Table S6-2. The input slacks and output slacks among hospitals between 2012 and 2021.

| Year | $I_{1}$ | $I_{2}$ | $I_{3}$ | $I_{4}$ | $I_{5}$ | $O_{1}$ | $O_{2}$ | $O_{3}$ | $O_{4}$ | $O_{5}$ |
| --- | --- | --- | --- | --- | --- | --- | --- | --- | --- | --- |
| 2012 | 0.353 | 8.653 | 1.695 | 18.046 | 10.639 | 518.928 | 203.326 | 591.100 | 1.035 | 0.586 |
| 2013 | 0.325 | 7.649 | 0.985 | 21.322 | 11.439 | 472.610 | 159.485 | 649.679 | 1.541 | 0.367 |
| 2014 | 0.282 | 3.412 | 0.210 | 16.563 | 5.246 | 293.838 | 19.663 | 236.855 | 0.931 | 0.204 |
| 2015 | 0.380 | 6.514 | 1.203 | 24.544 | 6.613 | 548.635 | 94.500 | 713.146 | 4.254 | 0.246 |
| 2016 | 0.580 | 7.156 | 0.286 | 23.403 | 11.876 | 558.312 | 123.919 | 1107.983 | 3.444 | 0.035 |
| 2017 | 0.488 | 9.796 | 1.067 | 20.318 | 8.532 | 454.098 | 144.561 | 995.136 | 3.638 | 0.058 |
| 2018 | 0.662 | 9.901 | 4.254 | 29.173 | 12.721 | 790.506 | 187.965 | 1799.549 | 2.784 | 0.045 |
| 2019 | 0.590 | 7.951 | 2.452 | 34.622 | 12.948 | 653.905 | 97.224 | 1447.325 | 3.915 | 0.048 |
| 2021 | 0.676 | 11.292 | 12.314 | 58.063 | 7.999 | 614.817 | 165.431 | 878.014 | 6.436 | 0.111 |
| Mean | 0.482 | 8.036 | 2.719 | 27.340 | 9.779 | 545.072 | 132.897 | 935.421 | 3.109 | 0.189 |

Table S7-1. Regional differences of medical institution efficiency value and its standard deviation in 2012-2021.

| Districts | Regions | 2012 | 2013 | 2014 | 2015 | 2016 | 2017 | 2018 | 2019 | 2021 | Mean |
| --- | --- | --- | --- | --- | --- | --- | --- | --- | --- | --- | --- |
| Eastern  districts | Beijing | 0.915 | 0.960 | 0.970 | 1.000 | 1.000 | 1.000 | 1.000 | 1.000 | 1.000 | 0.983 |
|  | Tianjin | 1.000 | 1.000 | 1.000 | 1.000 | 1.000 | 0.946 | 0.944 | 0.964 | 0.947 | 0.978 |
|  | Hebei | 1.000 | 1.000 | 1.000 | 0.928 | 0.928 | 0.901 | 0.888 | 0.903 | 0.864 | 0.935 |
|  | Liaoning | 0.860 | 0.861 | 0.881 | 0.865 | 0.862 | 0.854 | 0.851 | 0.853 | 0.824 | 0.857 |
|  | shanghai | 1.000 | 1.000 | 1.000 | 1.000 | 1.000 | 1.000 | 0.978 | 1.000 | 1.000 | 0.998 |
|  | Jiangsu | 1.000 | 1.000 | 1.000 | 1.000 | 0.990 | 1.000 | 0.991 | 1.000 | 1.000 | 0.998 |
|  | Zhejiang | 1.000 | 1.000 | 1.000 | 0.983 | 0.987 | 0.987 | 0.988 | 1.000 | 1.000 | 0.994 |
|  | Fujian | 1.000 | 1.000 | 1.000 | 1.000 | 1.000 | 1.000 | 1.000 | 1.000 | 1.000 | 1.000 |
|  | Shandong | 0.955 | 0.928 | 0.938 | 0.912 | 0.913 | 0.909 | 0.897 | 0.898 | 0.887 | 0.915 |
|  | Guangdong | 1.000 | 1.000 | 1.000 | 1.000 | 0.984 | 0.971 | 0.970 | 1.000 | 1.000 | 0.992 |
|  | Hainan | 1.000 | 1.000 | 0.946 | 0.925 | 0.931 | 0.927 | 0.921 | 1.000 | 0.925 | 0.953 |
|  | Mean | 0.976 | 0.977 | 0.976 | 0.965 | 0.963 | 0.954 | 0.948 | 0.965 | 0.950 | 0.964 |
|  | Standard deviation | 0.045 | 0.043 | 0.037 | 0.046 | 0.045 | 0.048 | 0.049 | 0.052 | 0.062 | 0.043 |
| Central districts | Shanxi | 0.885 | 0.885 | 0.889 | 0.870 | 0.885 | 0.878 | 0.879 | 0.909 | 0.851 | 0.881 |
|  | Jilin | 0.884 | 0.876 | 0.872 | 0.852 | 0.848 | 0.847 | 0.832 | 0.844 | 0.835 | 0.854 |
|  | Heilongjiang | 0.874 | 0.879 | 0.873 | 0.854 | 0.858 | 0.849 | 0.845 | 0.859 | 0.833 | 0.858 |
|  | Anhui | 1.000 | 1.000 | 1.000 | 1.000 | 1.000 | 1.000 | 1.000 | 1.000 | 0.934 | 0.993 |
|  | Jiangxi | 1.000 | 1.000 | 1.000 | 0.986 | 1.000 | 0.981 | 1.000 | 1.000 | 1.000 | 0.996 |
|  | Henan | 1.000 | 1.000 | 1.000 | 0.987 | 0.975 | 0.950 | 0.947 | 1.000 | 0.935 | 0.977 |
|  | Hubei | 0.963 | 0.983 | 1.000 | 0.954 | 0.950 | 0.940 | 0.958 | 1.000 | 1.000 | 0.972 |
|  | Hunan | 0.969 | 1.000 | 1.000 | 0.978 | 1.000 | 1.000 | 1.000 | 1.000 | 1.000 | 0.994 |
|  | Mean | 0.947 | 0.953 | 0.954 | 0.935 | 0.939 | 0.931 | 0.933 | 0.952 | 0.924 | 0.941 |
|  | Standard deviation | 0.053 | 0.057 | 0.059 | 0.061 | 0.062 | 0.060 | 0.066 | 0.065 | 0.070 | 0.060 |
| Western districts | Inner Mongolia | 0.885 | 0.879 | 0.874 | 0.846 | 0.848 | 0.836 | 0.838 | 0.846 | 0.819 | 0.852 |
|  | Guangxi | 1.000 | 1.000 | 1.000 | 0.955 | 0.959 | 0.948 | 0.947 | 1.000 | 0.957 | 0.974 |
|  | Chongqing | 0.971 | 1.000 | 1.000 | 0.963 | 1.000 | 1.000 | 0.941 | 1.000 | 1.000 | 0.986 |
|  | Sichuan | 1.000 | 1.000 | 0.989 | 0.922 | 0.923 | 0.941 | 0.930 | 1.000 | 1.000 | 0.967 |
|  | Guizhou | 1.000 | 1.000 | 1.000 | 0.956 | 0.957 | 0.962 | 1.000 | 1.000 | 1.000 | 0.986 |
|  | Yunnan | 1.000 | 1.000 | 1.000 | 1.000 | 1.000 | 0.970 | 1.000 | 1.000 | 1.000 | 0.997 |
|  | Tibet | 1.000 | 1.000 | 0.935 | 1.000 | 1.000 | 1.000 | 0.899 | 1.000 | 1.000 | 0.982 |
|  | Shaanxi | 1.000 | 0.946 | 0.933 | 0.924 | 0.914 | 0.916 | 0.917 | 0.925 | 1.000 | 0.942 |
|  | Gansu | 1.000 | 1.000 | 1.000 | 0.951 | 0.947 | 0.931 | 1.000 | 1.000 | 0.947 | 0.975 |
|  | Qinghai | 0.903 | 0.914 | 0.943 | 0.911 | 0.919 | 0.887 | 0.874 | 0.923 | 1.000 | 0.919 |
|  | Ningxia | 1.000 | 1.000 | 0.983 | 0.987 | 0.981 | 0.972 | 0.952 | 0.983 | 0.939 | 0.977 |
|  | Xinjiang | 0.949 | 0.955 | 1.000 | 0.963 | 0.908 | 0.961 | 0.934 | 1.000 | 0.885 | 0.951 |
|  | Mean | 0.976 | 0.975 | 0.971 | 0.948 | 0.946 | 0.944 | 0.936 | 0.973 | 0.962 | 0.959 |
|  | Standard deviation | 0.040 | 0.040 | 0.039 | 0.041 | 0.044 | 0.045 | 0.048 | 0.047 | 0.056 | 0.038 |

Table S7-2. Regional differences of hospital efficiency value and its standard deviation in 2012-2021.

| Districts | Regions | 2012 | 2013 | 2014 | 2015 | 2016 | 2017 | 2018 | 2019 | 2021 | Mean |
| --- | --- | --- | --- | --- | --- | --- | --- | --- | --- | --- | --- |
| Eastern districts | Beijing | 0.867 | 0.904 | 1.000 | 0.929 | 1.000 | 1.000 | 0.912 | 1.000 | 1.000 | 0.957 |
|  | Tianjin | 0.938 | 1.000 | 1.000 | 0.900 | 0.889 | 0.876 | 0.833 | 0.889 | 1.000 | 0.925 |
|  | Hebei | 0.922 | 1.000 | 1.000 | 0.903 | 0.952 | 0.925 | 0.886 | 0.891 | 0.874 | 0.928 |
|  | Liaoning | 0.835 | 0.867 | 0.938 | 0.929 | 0.893 | 0.909 | 0.870 | 0.855 | 0.880 | 0.886 |
|  | shanghai | 0.940 | 1.000 | 1.000 | 0.978 | 1.000 | 1.000 | 1.000 | 1.000 | 1.000 | 0.991 |
|  | Jiangsu | 0.911 | 0.929 | 0.961 | 0.935 | 0.935 | 0.948 | 0.913 | 0.930 | 0.932 | 0.933 |
|  | Zhejiang | 1.000 | 0.952 | 1.000 | 1.000 | 1.000 | 1.000 | 1.000 | 1.000 | 1.000 | 0.995 |
|  | Fujian | 1.000 | 0.965 | 0.973 | 0.926 | 0.933 | 0.959 | 0.927 | 0.928 | 0.951 | 0.951 |
|  | Shandong | 0.864 | 0.852 | 0.923 | 0.917 | 0.942 | 0.938 | 0.909 | 0.896 | 0.928 | 0.908 |
|  | Guangdong | 1.000 | 1.000 | 1.000 | 1.000 | 0.972 | 1.000 | 0.952 | 1.000 | 1.000 | 0.992 |
|  | Hainan | 0.836 | 0.831 | 0.860 | 0.823 | 0.825 | 0.834 | 0.827 | 0.837 | 0.821 | 0.833 |
|  | Mean | 0.919 | 0.936 | 0.969 | 0.931 | 0.940 | 0.944 | 0.912 | 0.930 | 0.944 | 0.936 |
|  | Standard deviation | 0.060 | 0.061 | 0.043 | 0.048 | 0.052 | 0.053 | 0.055 | 0.059 | 0.061 | 0.047 |
| Central districts | Shanxi | 0.784 | 0.801 | 0.826 | 0.789 | 0.792 | 0.808 | 0.789 | 0.784 | 0.814 | 0.799 |
|  | Jilin | 0.853 | 0.881 | 0.908 | 0.868 | 0.847 | 0.863 | 0.836 | 0.848 | 0.818 | 0.858 |
|  | Heilongjiang | 0.819 | 0.855 | 0.882 | 0.867 | 0.866 | 0.877 | 0.860 | 0.870 | 0.777 | 0.853 |
|  | Anhui | 0.932 | 0.935 | 1.000 | 0.957 | 0.950 | 1.000 | 0.960 | 1.000 | 0.939 | 0.964 |
|  | Jiangxi | 1.000 | 1.000 | 1.000 | 0.947 | 0.930 | 0.930 | 0.933 | 0.948 | 0.951 | 0.960 |
|  | Henan | 0.912 | 0.914 | 1.000 | 0.936 | 0.904 | 0.908 | 0.893 | 0.931 | 1.000 | 0.933 |
|  | Hubei | 1.000 | 1.000 | 1.000 | 1.000 | 0.982 | 1.000 | 1.000 | 1.000 | 1.000 | 0.998 |
|  | Hunan | 0.944 | 1.000 | 1.000 | 1.000 | 0.964 | 1.000 | 0.927 | 0.930 | 1.000 | 0.974 |
|  | Mean | 0.905 | 0.923 | 0.952 | 0.921 | 0.904 | 0.923 | 0.900 | 0.914 | 0.912 | 0.917 |
|  | Standard deviation | 0.075 | 0.070 | 0.065 | 0.069 | 0.061 | 0.068 | 0.065 | 0.070 | 0.088 | 0.067 |
| Western districts | Inner Mongolia | 0.834 | 0.827 | 0.849 | 0.813 | 0.804 | 0.820 | 0.801 | 0.817 | 0.816 | 0.834 |
|  | Guangxi | 1.000 | 1.000 | 1.000 | 1.000 | 1.000 | 1.000 | 1.000 | 1.000 | 1.000 | 1.000 |
|  | Chongqing | 0.922 | 0.935 | 0.979 | 0.975 | 0.968 | 0.959 | 0.914 | 0.932 | 0.968 | 0.922 |
|  | Sichuan | 1.000 | 1.000 | 1.000 | 0.965 | 0.929 | 1.000 | 0.906 | 0.931 | 0.946 | 1.000 |
|  | Guizhou | 1.000 | 1.000 | 1.000 | 0.960 | 0.964 | 1.000 | 1.000 | 1.000 | 1.000 | 1.000 |
|  | Yunnan | 1.000 | 1.000 | 1.000 | 1.000 | 1.000 | 1.000 | 1.000 | 1.000 | 1.000 | 1.000 |
|  | Tibet | 1.000 | 0.844 | 1.000 | 1.000 | 0.785 | 0.789 | 0.730 | 0.729 | 0.708 | 1.000 |
|  | Shaanxi | 0.872 | 0.910 | 1.000 | 0.965 | 0.942 | 1.000 | 0.960 | 0.943 | 1.000 | 0.872 |
|  | Gansu | 1.000 | 1.000 | 1.000 | 1.000 | 1.000 | 1.000 | 1.000 | 0.952 | 0.921 | 1.000 |
|  | Qinghai | 0.816 | 0.830 | 0.858 | 0.812 | 0.816 | 0.807 | 0.796 | 0.801 | 0.794 | 0.816 |
|  | Ningxia | 0.866 | 0.884 | 0.911 | 0.865 | 0.867 | 0.878 | 0.868 | 0.910 | 0.876 | 0.866 |
|  | Xinjiang | 1.000 | 0.981 | 1.000 | 1.000 | 1.000 | 1.000 | 0.978 | 1.000 | 0.969 | 1.000 |
|  | Mean | 0.943 | 0.934 | 0.966 | 0.946 | 0.923 | 0.938 | 0.913 | 0.918 | 0.917 | 0.943 |
|  | Standard deviation | 0.072 | 0.069 | 0.056 | 0.070 | 0.079 | 0.084 | 0.091 | 0.086 | 0.093 | 0.070 |

Table S8-1. Correlation coefficient between inputs and outputs among medical institutions.

|  | Number of outpatient visits  (1) | Number of hospitalizations  (2) | Number of inpatient surgery volume  (3) | Total income  (4) | in-hospital mortality rate  (5) |
| --- | --- | --- | --- | --- | --- |
| Number of medical institutions | 0.724 | 0.840 | 0.700 | 0.581 | -0.188 |
|  | (0.000) | (0.000) | (0.000) | (0.000) | (0.001) |
| Number of doctors | -0.005 | -0.054 | 0.174 | 0.296 | 0.404 |
|  | (0.936) | (0.347) | (0.002) | (0.000) | (0.000) |
| Number of registered nurses | 0.105 | 0.063 | 0.288 | 0.397 | 0.366 |
|  | (0.064) | (0.266) | (0.000) | (0.000) | (0.000) |
| Number of beds | -0.029 | 0.177 | 0.224 | 0.189 | 0.204 |
|  | (0.610) | (0.002) | (0.000) | (0.001) | (0.000) |
| Total expenditure | 0.897 | 0.765 | 0.921 | 0.993 | 0.254 |
|  | (0.000) | (0.000) | (0.000) | (0.000) | (0.000) |

Notes: Correlation coefficients and its p value (in parentheses) were reported.

Table S8-2. Correlation coefficient between inputs and outputs among hospitals.

|  | Number of outpatient visits  (1) | Number of hospitalizations  (2) | Number of inpatient surgery volume  (3) | Bed occupation rate  (4) | Average LOS  (5) |
| --- | --- | --- | --- | --- | --- |
| Number of medical institutions | 0.706 | 0.879 | 0.822 | 0.061 | -0.198 |
|  | (0.000) | (0.000) | (0.000) | (0.191) | (0.000) |
| Number of doctors | 0.315 | 0.213 | 0.294 | -0.228 | -0.193 |
|  | (0.000) | (0.000) | (0.000) | (0.000) | (0.000) |
| Number of registered nurses | 0.396 | 0.317 | 0.394 | -0.162 | -0.269 |
|  | (0.000) | (0.000) | (0.000) | (0.001) | (0.000) |
| Number of beds | 0.274 | 0.314 | 0.334 | -0.232 | -0.277 |
|  | (0.000) | (0.000) | (0.000) | (0.000) | (0.000) |
| Per-episode inpatient costs | 0.466 | 0.177 | 0.325 | 0.055 | 0.052 |
|  | (0.000) | (0.000) | (0.000) | (0.236) | (0.264) |

Notes: Correlation coefficients and its p value (in parentheses) were reported.

Table S9-1. Composition of average TFP changes in medical institutions across regions from 2012 to 2021.

| Regions | GML | TC | EC |
| --- | --- | --- | --- |
| Qinghai | 1.052 | 1.017 | 1.034 |
| Beijing | 1.045 | 1.045 | 1.000 |
| Hubei | 1.019 | 1.019 | 1.000 |
| Hunan | 1.016 | 1.016 | 1.000 |
| Chongqing | 1.015 | 1.015 | 1.000 |
| Zhejiang | 1.000 | 1.000 | 1.000 |
| Jiangxi | 1.000 | 1.000 | 1.000 |
| Sichuan | 1.000 | 1.000 | 1.000 |
| Fujian | 1.000 | 1.000 | 1.000 |
| Jiangsu | 1.000 | 1.000 | 1.000 |
| Shanghai | 1.000 | 1.000 | 1.000 |
| Tibet | 1.000 | 1.000 | 1.000 |
| Yunnan | 1.000 | 1.000 | 1.000 |
| Guangdong | 1.000 | 1.000 | 1.000 |
| Shanxi | 1.000 | 1.000 | 1.000 |
| Guizhou | 1.000 | 1.000 | 1.000 |
| Shanxi | 0.981 | 0.981 | 1.000 |
| Liaoning | 0.979 | 0.984 | 0.994 |
| Guangxi | 0.978 | 0.978 | 1.000 |
| Heilongjiang | 0.976 | 0.913 | 1.070 |
| Gansu | 0.973 | 0.973 | 1.000 |
| Tianjin | 0.973 | 0.973 | 1.000 |
| Jilin | 0.971 | 1.059 | 0.917 |
| Ningxia | 0.969 | 0.969 | 1.000 |
| Henan | 0.967 | 0.967 | 1.000 |
| Anhui | 0.966 | 0.966 | 1.000 |
| Xinjiang | 0.966 | 0.996 | 0.970 |
| Shandong | 0.964 | 1.004 | 0.960 |
| Hainan | 0.962 | 0.962 | 1.000 |
| Inner Mongolia | 0.962 | 1.013 | 0.949 |
| Hebei | 0.929 | 0.929 | 1.000 |

Abbreviations: TFP, total factor production efficiency. GML, global Malmquist_luenberger indexes. TC, technical change indexes. EC, efficiency change indexes.

Notes: GML=TC*EC.

Table S9-2. Composition of average TFP changes in hospitals across regions from 2012 to 2021.

| Regions | GML | TC | EC |
| --- | --- | --- | --- |
| Beijing | 1.074 | 1.074 | 1.000 |
| Shaanxi | 1.071 | 1.071 | 1.000 |
| Henan | 1.047 | 1.047 | 1.000 |
| Shandong | 1.037 | 1.035 | 1.002 |
| Tianjin | 1.033 | 1.033 | 1.000 |
| Shanghai | 1.031 | 1.031 | 1.000 |
| Hunan | 1.029 | 1.029 | 1.000 |
| Liaoning | 1.027 | 1.090 | 0.942 |
| Chongqing | 1.024 | 1.024 | 1.000 |
| Shanxi | 1.019 | 1.011 | 1.008 |
| Jiangsu | 1.012 | 1.018 | 0.993 |
| Ningxia | 1.006 | 1.006 | 1.000 |
| Anhui | 1.004 | 0.982 | 1.022 |
| Zhejiang | 1.000 | 1.000 | 1.000 |
| Hubei | 1.000 | 1.000 | 1.000 |
| Guizhou | 1.000 | 1.000 | 1.000 |
| Guangxi | 1.000 | 1.000 | 1.000 |
| Yunnan | 1.000 | 1.000 | 1.000 |
| Guangdong | 1.000 | 1.000 | 1.000 |
| Hainan | 0.991 | 0.981 | 1.010 |
| Inner Mongolia | 0.989 | 1.010 | 0.980 |
| Qinghai | 0.986 | 1.007 | 0.980 |
| Xinjiang | 0.985 | 0.985 | 1.000 |
| Jilin | 0.979 | 1.076 | 0.910 |
| Fujian | 0.975 | 0.975 | 1.000 |
| Jiangxi | 0.975 | 0.975 | 1.000 |
| Heilongjiang | 0.974 | 1.026 | 0.949 |
| Hebei | 0.973 | 0.935 | 1.041 |
| Sichuan | 0.973 | 1.000 | 0.973 |
| Gansu | 0.960 | 0.981 | 0.978 |
| Tibet | 0.841 | 0.841 | 1.000 |

Abbreviations: TFP, total factor production efficiency. GML, global Malmquist_luenberger indexes. TC, technical change indexes. EC, efficiency change indexes.

Notes: GML=TC*EC.

Table S10. Average TFP changes of medical service and its decomposition in each group between 2012 and 2021.

|  | Medical institutions | | | Hospitals | | |
| --- | --- | --- | --- | --- | --- | --- |
|  | GML | TC | EC | GML | TC | EC |
| 31 regions as a whole | 0.990 | 0.995 | 0.998 | 1.002 | 1.009 | 0.994 |
| Primary services volume | | | | | | |
| Low-level group (Q1) | 0.996 | 0.990 | 1.011 | 1.016 | 1.034 | 0.985 |
| Middle-level group (Q2, Q3) | 0.995 | 1.005 | 0.993 | 0.997 | 1.004 | 0.994 |
| High-level group (Q4) | 0.972 | 0.978 | 0.994 | 0.997 | 0.994 | 1.003 |
| Primary medical staff | | | | | | |
| Low-level group (Q1) | 0.996 | 0.990 | 1.011 | 1.016 | 1.034 | 0.985 |
| Middle-level group (Q2, Q3) | 0.982 | 0.994 | 0.991 | 0.995 | 0.998 | 0.997 |
| High-level group (Q4) | 1.002 | 1.002 | 1.000 | 1.003 | 1.008 | 0.996 |
| GDP | | | | | | |
| Low-level group (Q1) | 0.989 | 1.001 | 0.992 | 0.971 | 0.987 | 0.985 |
| Middle-level group (Q2, Q3) | 0.989 | 0.990 | 1.003 | 1.014 | 1.018 | 0.997 |
| High-level group (Q4) | 0.993 | 0.999 | 0.994 | 1.011 | 1.015 | 0.996 |

Abbreviations: GML, global Malmquist_luenberger indexes. TC, technical change indexes. EC, efficiency change indexes.

Notes: GML=TC*EC. Average TFP changes and its decomposition is sorted by three grouping criteria, namely the proportion of primary service volume, the proportion of primary medical staff, and the gross regional product ([GDP](https://baike.baidu.com/item/GDP/41201?fromModule=lemma_inlink)).

Table S11. Average TFP changes and its decomposition of medical service in China (after adding 2020 data).

|  | Medical institutions | | | | | |  |  | Hospitals | | | | |
| --- | --- | --- | --- | --- | --- | --- | --- | --- | --- | --- | --- | --- | --- |
| Year | Efficiency scores | GML |  | TC |  | EC |  | Efficiency scores | GML |  | TC |  | EC |
| 2012 | 0.915 |  |  |  |  |  |  | 0.973 |  |  |  |  |  |
| 2013 | 0.938 | 1.006 |  | 1.002 |  | 1.004 |  | 0.979 | 1.026 |  | 1.018 |  | 1.008 |
| 2014 | 0.957 | 0.996 |  | 0.997 |  | 1.000 |  | 0.975 | 1.021 |  | 1.019 |  | 1.002 |
| 2015 | 0.886 | 0.947 |  | 0.956 |  | 0.991 |  | 0.924 | 0.926 |  | 0.941 |  | 0.985 |
| 2016 | 0.918 | 1.013 |  | 1.007 |  | 1.006 |  | 0.935 | 1.036 |  | 1.033 |  | 1.004 |
| 2017 | 0.920 | 0.991 |  | 0.992 |  | 0.999 |  | 0.927 | 1.002 |  | 1.010 |  | 0.993 |
| 2018 | 0.899 | 1.005 |  | 1.008 |  | 0.997 |  | 0.932 | 0.979 |  | 0.978 |  | 1.002 |
| 2019 | 0.919 | 1.023 |  | 1.030 |  | 0.994 |  | 0.953 | 1.022 |  | 1.049 |  | 0.976 |
| 2020 | 0.862 | 0.871 |  | 0.861 |  | 1.014 |  | 0.831 | 0.941 |  | 0.945 |  | 0.996 |
| 2021 | 0.893 | 1.132 |  | 1.134 |  | 0.998 |  | 0.935 | 1.039 |  | 1.019 |  | 1.020 |
| Mean | 0.911 | 0.982 |  | 0.983 |  | 1.001 |  | 0.936 | 0.993 |  | 1.002 |  | 0.993 |

Abbreviations: GML, global Malmquist_luenberger indexes. TC, technical change indexes. EC, efficiency change indexes.

Notes: GML=TC*EC.

Table S12. Changes in average TFP and the decomposition of medical services in China by excluding the 2020 and 2021 data.

| Year | Medical institutions | | | | Hospitals | | | |
| --- | --- | --- | --- | --- | --- | --- | --- | --- |
|  | Efficiency scores | GML | TC | EC | Efficiency scores | GML | TC | EC |
| 2012-2021 | 0.936 | 0.982 | 0.983 | 1.001 | 0.911 | 0.993 | 1.002 | 0.993 |
| 2012-2021  (Excluding 2020 data only) | 0.956 | 0.990 | 0.995 | 0.998 | 0.930 | 1.002 | 1.009 | 0.994 |
| 2012-2019 | 0.956 | 1.000 | 1.001 | 0.999 | 0.930 | 1.002 | 1.002 | 1.000 |

Abbreviations: GML, global Malmquist_luenberger indexes. TC, technical change indexes. EC, efficiency change indexes.

Notes: GML=TC*EC.

Table S13. Relative efficiency of medical institutions in 31 Chinese regions from 2012 to 2021.

| Regions | ${TE}_{CRS}$ | ${TE}_{VRS}$ | SE |
| --- | --- | --- | --- |
| Anhui | 1.000 | 1.000 | 1.000 |
| Beijing | 1.000 | 1.000 | 1.000 |
| Chongqing | 1.000 | 1.000 | 1.000 |
| Fujian | 1.000 | 1.000 | 1.000 |
| Gansu | 1.000 | 1.000 | 1.000 |
| Guangdong | 1.000 | 1.000 | 1.000 |
| Guangxi | 1.000 | 1.000 | 1.000 |
| Guizhou | 1.000 | 1.000 | 1.000 |
| Hainan | 0.983 | 0.996 | 0.986 |
| Hebei | 1.000 | 1.000 | 1.000 |
| Heilongjiang | 1.000 | 1.000 | 1.000 |
| Henan | 1.000 | 1.000 | 1.000 |
| Hubei | 1.000 | 1.000 | 1.000 |
| Hunan | 0.991 | 0.999 | 0.992 |
| Inner Mongolia | 0.995 | 0.997 | 0.998 |
| Jiangsu | 0.991 | 0.991 | 0.999 |
| Jiangxi | 1.000 | 1.000 | 1.000 |
| Jilin | 1.000 | 1.000 | 1.000 |
| Liaoning | 0.985 | 0.996 | 0.990 |
| Ningxia | 0.954 | 0.955 | 0.999 |
| Qinghai | 0.985 | 0.990 | 0.995 |
| Shaanxi | 0.994 | 1.000 | 0.994 |
| Shandong | 0.978 | 1.000 | 0.978 |
| shanghai | 1.000 | 1.000 | 1.000 |
| Shanxi | 0.939 | 0.942 | 0.997 |
| Sichuan | 1.000 | 1.000 | 1.000 |
| Tianjin | 1.000 | 1.000 | 1.000 |
| Tibet | 1.000 | 1.000 | 1.000 |
| Xinjiang | 0.995 | 1.000 | 0.995 |
| Yunnan | 1.000 | 1.000 | 1.000 |
| Zhejiang | 1.000 | 1.000 | 1.000 |
| Mean | 0.993 | 0.996 | 0.997 |

Abbreviations: CRS, constant return to scale. VRS, variable return to scale. DEA, data envelopment analysis. ${TE}_{CRS}$, overall technical efficiency from CRS DEA. ${TE}_{VRS}$, pure technical efficiency from VRS DEA. SE, scale efficiency =${TE}_{CRS}$ / ${TE}_{VRS}$.

Table S14. Relative efficiency of hospitals in 31 Chinese regions from 2012 to 2021.

| Regions | ${TE}_{CRS}$ | ${TE}_{VRS}$ | SE |
| --- | --- | --- | --- |
| Anhui | 0.979 | 0.979 | 1.000 |
| Beijing | 0.953 | 1.000 | 0.953 |
| Chongqing | 1.000 | 1.000 | 1.000 |
| Fujian | 1.000 | 1.000 | 1.000 |
| Gansu | 1.000 | 1.000 | 1.000 |
| Guangdong | 1.000 | 1.000 | 1.000 |
| Guangxi | 1.000 | 1.000 | 1.000 |
| Guizhou | 1.000 | 1.000 | 1.000 |
| Hainan | 0.888 | 0.948 | 0.937 |
| Hebei | 0.986 | 0.992 | 0.994 |
| Heilongjiang | 0.847 | 1.000 | 0.847 |
| Henan | 1.000 | 1.000 | 1.000 |
| Hubei | 1.000 | 1.000 | 1.000 |
| Hunan | 1.000 | 1.000 | 1.000 |
| Inner Mongolia | 0.811 | 0.947 | 0.857 |
| Jiangsu | 0.945 | 0.986 | 0.959 |
| Jiangxi | 1.000 | 1.000 | 1.000 |
| Jilin | 0.787 | 0.931 | 0.846 |
| Liaoning | 0.792 | 1.000 | 0.792 |
| Ningxia | 0.889 | 0.982 | 0.906 |
| Qinghai | 0.826 | 0.991 | 0.833 |
| Shaanxi | 0.891 | 0.964 | 0.925 |
| Shandong | 0.976 | 1.000 | 0.976 |
| shanghai | 1.000 | 1.000 | 1.000 |
| Shanxi | 0.861 | 1.000 | 0.861 |
| Sichuan | 1.000 | 1.000 | 1.000 |
| Tianjin | 0.995 | 1.000 | 0.995 |
| Tibet | 1.000 | 1.000 | 1.000 |
| Xinjiang | 1.000 | 1.000 | 1.000 |
| Yunnan | 1.000 | 1.000 | 1.000 |
| Zhejiang | 1.000 | 1.000 | 1.000 |
| Mean | 0.949 | 0.991 | 0.957 |

Abbreviations: CRS, constant return to scale. VRS, variable return to scale. DEA, data envelopment analysis. ${TE}_{CRS}$, overall technical efficiency from CRS DEA. ${TE}_{VRS}$, pure technical efficiency from VRS DEA. SE, scale efficiency =${TE}_{CRS}$ / ${TE}_{VRS}$.

Table S15. Malmquist index summary of the medical institutions in China from 2012 to 2021 (output- oriented).

| Year | TFP | TC | TEC | PEC | SE |
| --- | --- | --- | --- | --- | --- |
| 2013-2012 | 0.982 | 0.983 | 0.999 | 1.000 | 0.999 |
| 2014-2013 | 0.995 | 0.995 | 1.000 | 0.999 | 1.001 |
| 2015-2014 | 0.977 | 0.979 | 0.998 | 0.997 | 1.000 |
| 2016-2015 | 0.994 | 0.997 | 0.997 | 0.998 | 1.000 |
| 2017-2016 | 0.994 | 0.994 | 1.000 | 1.003 | 0.997 |
| 2018-2017 | 0.983 | 0.98 | 1.003 | 1.000 | 1.003 |
| 2019-2018 | 1.008 | 1.008 | 1.000 | 0.999 | 1.001 |
| 2021-2020 | 0.93 | 0.937 | 0.993 | 0.998 | 0.994 |
| mean | 0.983 | 0.984 | 0.999 | 0.999 | 0.999 |

Abbreviations: TFP, total factor productivity. TC, technical change. TEC, technical efficiency change. PEC, pure technical efficiency. SE, scale efficiency.

Notes: TFP = TC × TEC =TC × (PTE × SE).

Table S16. Malmquist index summary of the hospitals in China from 2012 to 2021 (output- oriented).

| Year | TFP | TC | TEC | PEC | SE |
| --- | --- | --- | --- | --- | --- |
| 2013-2012 | 0.96 | 0.955 | 1.005 | 1.002 | 1.003 |
| 2014-2013 | 0.984 | 0.983 | 1.001 | 0.998 | 1.003 |
| 2015-2014 | 0.953 | 0.951 | 1.003 | 1.000 | 1.003 |
| 2016-2015 | 0.983 | 0.985 | 0.998 | 0.998 | 1.000 |
| 2017-2016 | 0.975 | 0.968 | 1.008 | 0.999 | 1.009 |
| 2018-2017 | 0.977 | 0.984 | 0.993 | 0.996 | 0.996 |
| 2019-2018 | 0.976 | 0.975 | 1.001 | 0.996 | 1.005 |
| 2021-2020 | 0.879 | 0.888 | 0.99 | 0.993 | 0.997 |
| mean | 0.960 | 0.961 | 1.000 | 0.998 | 1.002 |

Abbreviations: TFP, total factor productivity. TC, technical change. TEC, technical efficiency change. PEC, pure technical efficiency. SE, scale efficiency.

Notes: TFP = TC × TEC =TC × (PTE × SE).
