# Supplementary material for: Evaluation of healthcare efficiency in China: a three-stage data envelopment analysis of directional slacks-based measure
Source: Front Public Health. 2024 May 30;12:1393143. doi: 10.3389/fpubh.2024.1393143 (PMC11169848; doi:10.3389/fpubh.2024.1393143)
Supplement: Supplementary file 4 [file Data_Sheet_4.docx]

**Appendix B: Descriptions on the process of input-output adjustments**

**1. Input adjustments**

Medical institutions’ adjusted inputs are constructed from the results of SFA regressions by means of:

$$\begin{aligned} X_{ni}^{A}=X_{ni}+\left[ \max\left( f\left( P_{i};\hat{\beta}_{n} \right) \right)-f\left( P_{i};\hat{\beta}_{n} \right) \right]+\left[ \max\left( v_{ni} \right)-v_{ni} \right]; \\ i=1,2,\cdots I;n=1,2,\cdots,N.\#\left( B-1 \right) \end{aligned}$$

where $X_{ni}^{A}$ and $X_{ni}$ are adjusted inputs and observed inputs, respectively. $\left[ \max\left( f\left( P_{i};\hat{\beta}_{n} \right) \right)-f\left( P_{i};\hat{\beta}_{n} \right) \right]$ puts all medical institutions into a common operating environment, the least favorable environment observed in the sample. $\left[ \max\left( v_{ni} \right)-v_{ni} \right]$ puts all medical institutions into a common state of nature, the unluckiest situation encountered in the sample. Thus, medical institutions with relatively unfavorable operating environments or bad luck have their inputs adjusted upward by a relatively small amount, while medical institutions with relatively favorable operating environments or good luck have their inputs adjusted upward by a relatively large amount. These adjustments vary across both medical institutions and inputs (1).

**2. Output adjustments**

Adjusted desirable outputs of medical institutions are constructed from the results of SFA regressions by means of:

$$\begin{aligned} Y_{mi}^{A}=Y_{mi}+\left[ f\left( P_{i};\hat{\beta}_{n} \right)-\min\left( f\left( P_{i};\hat{\beta}_{n} \right) \right) \right]+\left[ v_{ni}-\min\left( v_{ni} \right) \right]; \\ i=1,2,\cdots I;n=1,2,\cdots,N.\#\left( B-2 \right) \end{aligned}$$

where $Y_{mi}^{A}$ and $Y_{mi}$ are adjusted outputs and observed outputs, respectively. $\left[ f\left( P_{i};\hat{\beta}_{n} \right)-\min\left( f\left( P_{i};\hat{\beta}_{n} \right) \right) \right]$ puts all medical institutions into a common operating environment, the most favorable environment observed in the sample. $\left[ v_{ni}-\min\left( v_{ni} \right) \right]$ puts all medical institutions into a common state of nature, the luckiest situation encountered in the sample.

To implement equation (B-1) and equation (B-2), it is necessary to separate statistical noise from managerial inefficiency in the residuals of SFA regression models to obtain estimates of $v_{ni}$ for each DMU. The first step was to separate managerial inefficiency $u_{ni}$, which was accomplished by using the methodology proposed by Jondrow et al. (1982). However, their paper used a production function, and the mixed error term was $\varepsilon=\nu-u$. In our SFA regression, which adopted a cost function, the form of the separation formula is as follows (2,3):

$$\begin{aligned} E(u|\varepsilon)=\sigma_{*}[\frac{\emptyset(\lambda\frac{\varepsilon}{\sigma})}{\Phi(\frac{\lambda\varepsilon}{\sigma})}+\frac{\lambda\varepsilon}{\sigma}].\#\left( B-3 \right) \end{aligned}$$

where $\sigma_{*}=\frac{\sigma_{u}\sigma_{v}}{\sigma}$, $\sigma=\sqrt{\sigma_{u}^{2}+\sigma_{v}^{2}}$, $\lambda=\sigma_{u}/\sigma_{v}$. $\sigma_{u}$ and $\sigma_{v}$ represent the standard deviation of managerial inefficiency and random error term, respectively. The mixed error term $\varepsilon$ denotes the difference between original input-output values and environmental values $f\left( P_{i};\beta_{n} \right)$. The second step is to calculate the random error term $\nu$, and the calculation formula is as follows:

$$\begin{aligned} E\left[ v_{ni} | v_{ni}+u_{ni} \right]=s_{ni}-f\left( z_{i};\beta_{n} \right)-E\left[ u_{ni} | v_{ni}+u_{ni} \right].\#\left( B-4 \right) \end{aligned}$$

Where $E\left[ v_{ni} | v_{ni}+u_{ni} \right]$ provide conditional (on $v_{ni}+u_{ni}$) estimates for the $v_{ni}$ in equation (B-1) and equation (B-2).

**References**

1. Fried HO, Lovell CAK, Schmidt SS, Yaisawarng S. Accounting for Environmental Effects and Statistical Noise in Data Envelopment Analysis. Journal of Productivity Analysis. 2002 Jan 1;17(1):157–74.

2. Luo D. A Note on Estimating Managerial Inefficiency of Three-Stage DEA Model. Statistical Research. 2012;29(4):104–7.

3. CHEN W wei, ZHANG L, MA T hu, LIU Q ling. Research on Three stage DEA Model. System Engineering. 2014;32(9):144–9.
