# Supplementary material for: Evaluation of healthcare efficiency in China: a three-stage data envelopment analysis of directional slacks-based measure
Source: Front Public Health. 2024 May 30;12:1393143. doi: 10.3389/fpubh.2024.1393143 (PMC11169848; doi:10.3389/fpubh.2024.1393143)
Supplement: Supplementary file 5 [file Data_Sheet_5.docx]

**Appendix C: Inefficiency value decomposition**

The inefficiency value calculated by Formula (2) is further decomposed to obtain the specific source of inefficiency:

$$\begin{aligned} IE=S_{v}^{t}={IE}_{v}^{x}+{IE}_{v}^{y}+{IE}_{v}^{b}.\#\left( C-1 \right) \end{aligned}$$

${IE}_{v}^{x}$, ${IE}_{v}^{y}$, and ${IE}_{v}^{b}$ represents the inefficiency values of input, desirable output and un desirable output, respectively. They can be calculated by the following formula:

$$\begin{aligned} {IE}_{v}^{x}=\frac{1}{3N}\sum_{n=1}^{N} \frac{S_{n}^{x}}{g_{n}^{x}}.\#\left( C-2 \right) \end{aligned}$$

$$\begin{aligned} {IE}_{v}^{y}=\frac{1}{3M}\sum_{m=1}^{N} \frac{S_{m}^{y}}{g_{m}^{y}}.\#\left( C-3 \right) \end{aligned}$$

$\begin{aligned} {IE}_{v}^{b}=\frac{1}{3J}\sum_{j=1}^{J} \frac{S_{j}^{b}}{g_{j}^{b}}.\#\left( C-4 \right) \end{aligned}$

The inputs of medical institutions in this study includes the per-unit-population number of medical institutions, doctors, registered nurses, and beds, as well as the total expenditure of medical institutions. The desirable outputs include the number of outpatient visits, the number of hospitalizations, inpatient surgery volume, and total income of medical institutions. The undesirable output includes in-hospital mortality. Therefore, input inefficiency ${IE}_{v}^{x}$, desirable output inefficiency ${IE}_{v}^{y}$, undesirable output inefficiency ${IE}_{v}^{b}$ can be further decomposed:

$$\begin{aligned} {IE}_{v}^{x\_medical institutions}={IE}_{v}^{number of medical institutions}+{IE}_{v}^{number of doctors} \\ +{IE}_{v}^{number of registered nurses}+{IE}_{v}^{number of beds}+{IE}_{v}^{total expenditure}.\#\left( C-5 \right) \end{aligned}$$

$$\begin{aligned} {IE}_{v}^{y\_medical institutions}={IE}_{v}^{number of outpatient visits}+{IE}_{v}^{number of \mathrm{hospitalizations}} \\ +{IE}_{v}^{inpatient surgery volume}+{IE}_{v}^{total income}.\#\left( C-6 \right) \end{aligned}$$

$$\begin{aligned} {IE}_{v}^{b\_medical institutions}={IE}_{v}^{\mathrm{in}-hospital mortality}.\#\left( C-7 \right) \end{aligned}$$

Similarly, the inputs of hospitals include the number of hospitals, doctors, registered nurses, and beds, as well as per-episode inpatient costs. As such, the input inefficiency of hospitals ${IE}_{v}^{x\_\mathrm{hospitals}}$ could be decomposed:

$$\begin{aligned} {IE}_{v}^{x\_\mathrm{hospitals}}={IE}_{v}^{number of hospitals}+{IE}_{v}^{number of doctors} \\ +{IE}_{v}^{number of registered nurses}+{IE}_{v}^{number of beds}+{IE}_{v}^{per-episode inpatient costs}.\#\left( C-8 \right) \end{aligned}$$

The number of outpatient visits, the number of hospitalizations, inpatient surgery volume, and occupancy rate of hospital beds were included as the desirable outputs of hospitals. The input inefficiency of hospitals ${IE}_{v}^{y\_\mathrm{hospitals}}$ could be decomposed:

$$\begin{aligned} {IE}_{v}^{y\_\mathrm{hospitals}}={IE}_{v}^{number of outpatient visits}+{IE}_{v}^{number of \mathrm{hospitalizations}} \\ +{IE}_{v}^{inpatient surgery volume}+{IE}_{v}^{occupancy rate of hospital beds}.\#\left( C-9 \right) \end{aligned}$$

The undesirable output of hospitals includes the average LOS,

$$\begin{aligned} {IE}_{v}^{b\_hospitals}={IE}_{v}^{average LOS}.\#\left( C-10 \right) \end{aligned}$$

Therefore, inefficiency value calculated by Formula (2) could be ultimately decomposed:

$$\begin{aligned} {IE}^{medical institutions}={IE}_{v}^{x_{\mathrm{medical}}\mathrm{institutions}}+{IE}_{v}^{y_{\mathrm{medical}}\mathrm{institutions}}+{IE}_{v}^{b_{\mathrm{medical}}\mathrm{institutions}} \\ ={IE}_{v}^{number of medical institutions}+{IE}_{v}^{number of doctors}+{IE}_{v}^{number of registered nurses} \\ +{IE}_{v}^{number of beds}+{IE}_{v}^{total expenditure}+{IE}_{v}^{number of outpatient visits}+{IE}_{v}^{number of \mathrm{hospitalizations}} \\ +{IE}_{v}^{inpatient surgery volume}+{IE}_{v}^{total income}+{IE}_{v}^{\mathrm{in}-hospital mortality}.\#\left( C-11 \right) \end{aligned}$$

$$\begin{aligned} {IE}^{\mathrm{hospitals}}={IE}_{v}^{x\_\mathrm{hospitals}}+{IE}_{v}^{y\_\mathrm{hospitals}}+{IE}_{v}^{b\_hospitals} \\ ={IE}_{v}^{number of \mathrm{hospitals}}+{IE}_{v}^{number of doctors}+{IE}_{v}^{number of registered nurses} \\ +{IE}_{v}^{number of beds}+{IE}_{v}^{per-episode inpatient costs}+{IE}_{v}^{number of outpatient visits} \\ +{IE}_{v}^{number of \mathrm{hospitalizations}}+{IE}_{v}^{inpatient surgery volume}+{IE}_{v}^{occupancy rate of hospital beds} \\ +{IE}_{v}^{\mathrm{in}-hospital mortality}.\#\left( C-12 \right) \end{aligned}$$
