# Supplementary figures and images for: Evaluation of healthcare efficiency in China: a three-stage data envelopment analysis of directional slacks-based measure
Source: Front Public Health. 2024 May 30;12:1393143. doi: 10.3389/fpubh.2024.1393143 (PMC11169848; doi:10.3389/fpubh.2024.1393143)

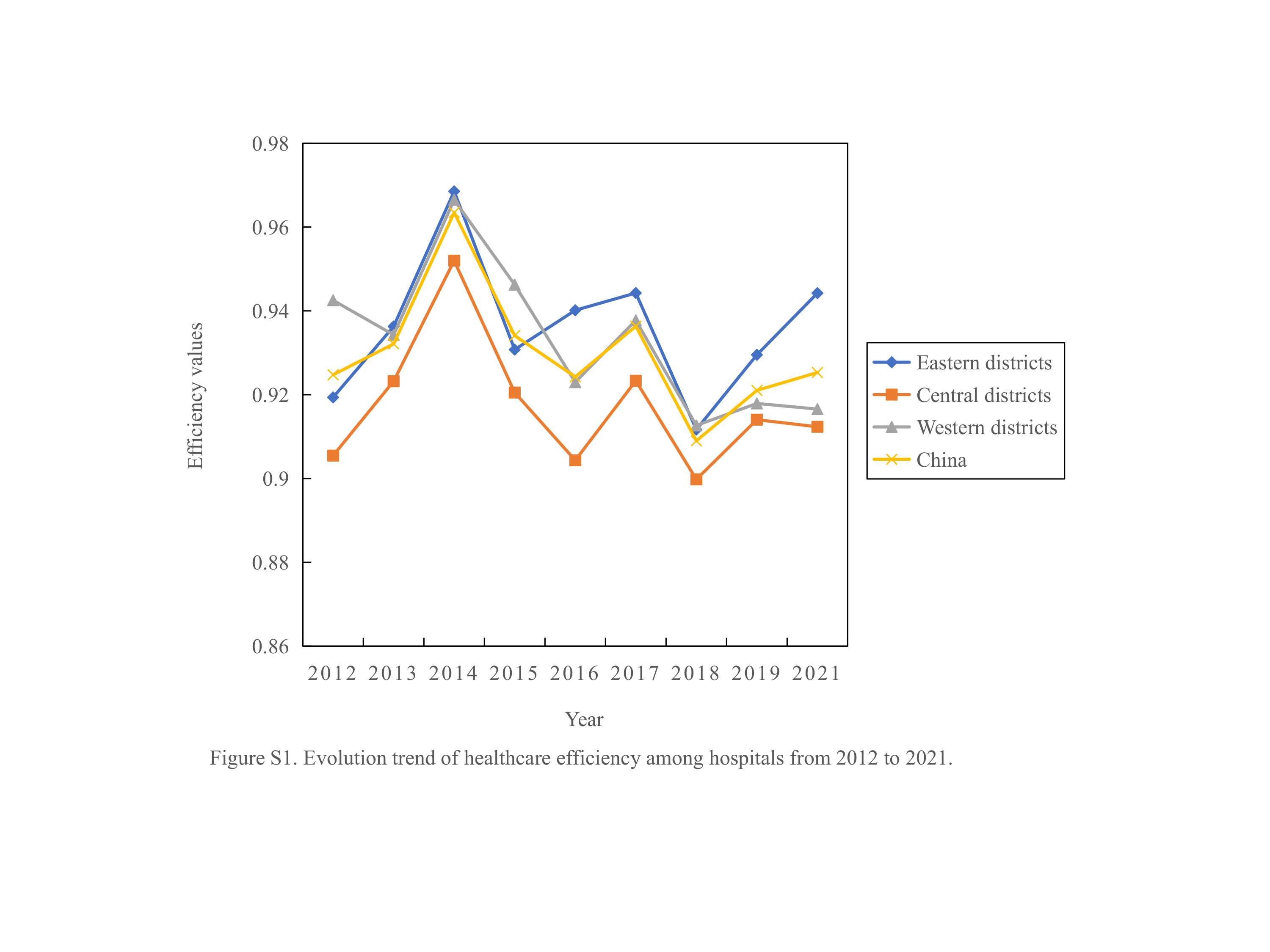

Supplement: Supplementary file 6 [file Image_1.TIF]
